# Complex Metal Borohydrides: From Laboratory Oddities to Prime Candidates in Energy Storage Applications

**DOI:** 10.3390/ma15062286

**Published:** 2022-03-19

**Authors:** Cezar Comanescu

**Affiliations:** 1 National Institute of Materials Physics, 405A Atomiștilor St., 077125 Magurele, Romania; cezar.comanescu@infim.ro; 2 Inorganic Chemistry Department, Politehnica University of Bucharest, 1 Polizu St., 011061 Bucharest, Romania; 3 Faculty of Physics, University of Bucharest, 405, Atomiștilor St., 077125 Magurele, Romania

**Keywords:** energy storage, metal borohydride, recyclability, kinetic destabilization, hydrogen

## Abstract

Despite being the lightest element in the periodic table, hydrogen poses many risks regarding its production, storage, and transport, but it is also the one element promising pollution-free energy for the planet, energy reliability, and sustainability. Development of such novel materials conveying a hydrogen source face stringent scrutiny from both a scientific and a safety point of view: they are required to have a high hydrogen wt.% storage capacity, must store hydrogen in a safe manner (i.e., by chemically binding it), and should exhibit controlled, and preferably rapid, absorption–desorption kinetics. Even the most advanced composites today face the difficult task of overcoming the harsh re-hydrogenation conditions (elevated temperature, high hydrogen pressure). Traditionally, the most utilized materials have been RMH (reactive metal hydrides) and complex metal borohydrides M(BH_4_)_x_ (M: main group or transition metal; x: valence of M), often along with metal amides or various additives serving as catalysts (Pd^2+^, Ti^4+^ etc.). Through destabilization (kinetic or thermodynamic), M(BH_4_)_x_ can effectively lower their dehydrogenation enthalpy, providing for a faster reaction occurring at a lower temperature onset. The present review summarizes the recent scientific results on various metal borohydrides, aiming to present the current state-of-the-art on such hydrogen storage materials, while trying to analyze the pros and cons of each material regarding its thermodynamic and kinetic behavior in hydrogenation studies.

## 1. Introduction

**Figure 1:**
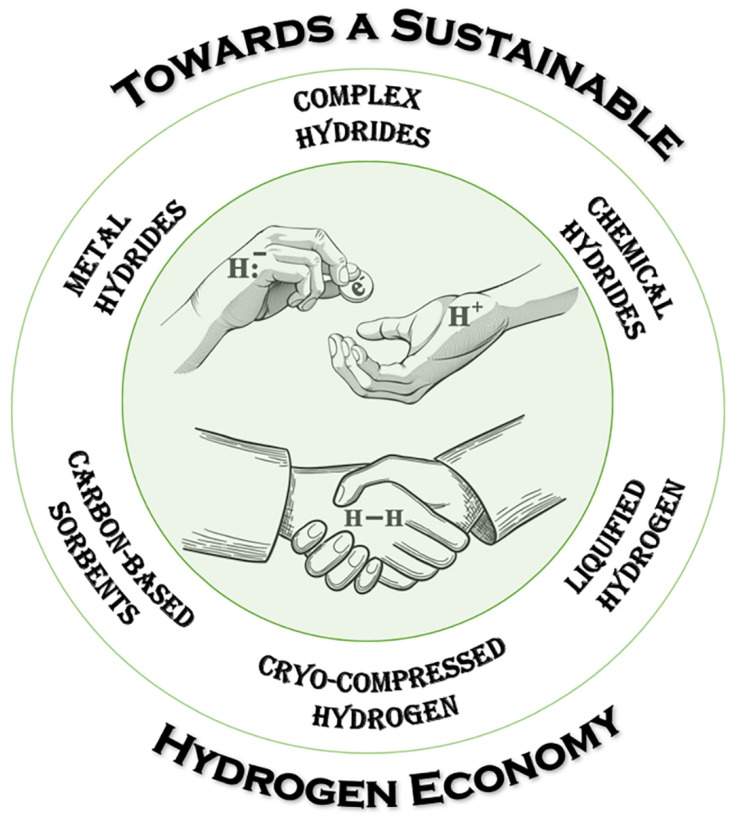
Convergence of hydrogen storage methods towards a sustainable hydrogen economy.

A world with ever more scarce fossil fuels must transition to alternative energy storing materials. Fossil fuels, such as coal, gas, and oil, are finite, non-renewable resources, and a current estimation based on their ongoing burning rate points to a complete depletion by the end of 2060. While they are more reliable than currently engineered materials, they also pollute the environment and are responsible in great part for the greenhouse effect [1,2,3,4,5,6,7,8,9]. While the risk of running out of fossil fuels has been advocated for decades, the timeline is approaching and humanity needs to make a change. Therefore, alternative energy carriers are long sought-after; among them, metal hydrides and complex borohydrides have a special role [10,11,12,13,14,15,16,17,18]. Reaching or exceeding the U.S. Department of Energy (DOE) target is no easy task; the specific target for 2020 was 1.5 kWh/kg (4.5 wt.% hydrogen) and 1.0 kWh/L (0.03 Kg hydrogen/L), costing more than USD 10 /kWh (or USD 333/Kg of stored hydrogen capacity) [19,20]. A series of options have been investigated for hydrogen storage applications: cryo-compression (7–27 Kg/m^3^), metal hydrides (40–70 Kg/m^3^), chemical hydrides (50–120 Kg/m^3^), carbon sorption (20–50 Kg/m^3^), complex hydrides, and liquified dihydrogen (70 Kg/m^3^) (Figure 1).

**Figure 2:**
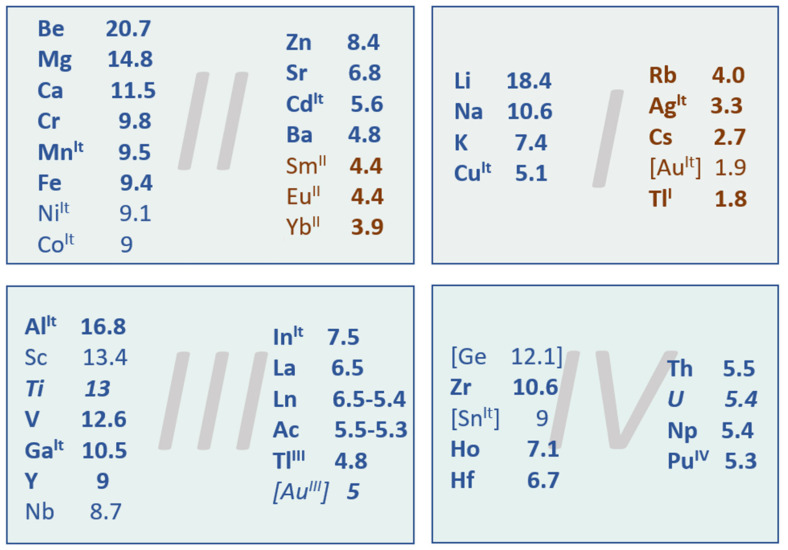
Theoretical (maximum) hydrogen storage capacity of metal borohydrides. Metal borohydrides are arranged by cation valence (I, II, III, and IV). Highlighted in blue are the borohydrides that exceed the DOE’s hydrogen storage capacity set for 2020 (4.5 wt %). **Ln** = La, Ce, Pr, Nd, Sm(II, III), Eu(II, III), Gd, Tb, Dy, Ho, Er, Tm, Yb(II, III), Lu; **Ac** = Th, Pa, *U*, Np, Pu; *^lt^*-isolable at low temperatures; in **bold** are shown borohydrides reported by several groups (higher confidence).

A special role is held by complex metal hydrides and borohydrides in particular, given their high gravimetric energy content (Figure 2).

Computing the theoretical hydrogen storage of a complex metal borohydride yields the following:

(1) $$\mathrm{wt}.\;\%\;\mathrm{hydrogen}\;\mathrm{in}\;M{\left({\mathrm{BH}}_{4}\right)}_{x}=\frac{4\;x\;}{A_{M}+14.842\;x}\times 100=\frac{400}{\frac{A_{M}}{x}+14.842}\;\%$$

This relation would imply that, in order to reach a minimum storage capacity set by the DOE of 4.5 wt.%, the following relation should hold:

(2) $$\frac{400}{\frac{A_{M}}{x}+14.842}\geq 4.5\;\Rightarrow \;\frac{A_{M}}{x}\leq 74.406$$

One could argue that the DOE target could be met, looking at Equations (1) and (2), by any mono-valent metal having *A_M_* $\leq 74.406\frac{g}{\mathrm{mol}}$, or a divalent metal with *A_M_* $\;\leq 148.812\;\frac{g}{\mathrm{mol}}$, or a trivalent metal with *A_M_* $\leq 223.218\;\frac{g}{\mathrm{mol}}$ a.s.o. Indeed, mTHRost known borohydrides fall into this category: they afford a theoretical hydrogen storage capacity superior to that imposed by the DOE (4.5 wt.%). Equation (2) also implies that the hydrogen storage capacity will be the highest for the most lightweight borohydrides, thus corresponding to low *A_M_* values: second, third, and fourth period metals are the borohydrides most used to date [21,22,23,24,25,26,27]. A summary of known borohydrides with specific hydrogen storage capacity is presented in Figure 2, which are arranged by metal valency and atomic weight of the metal, in decreasing order of their theoretical hydrogen storage capacity (wt.%). The maximum hydrogen storage capacity (denoted herein as: H, wt.%) is computed for a series of mono-, di-, tri-, and tetravalent metals; in some cases, only hypothetic formulae were used since the actual borohydride’s existence is unclear (AuBH_4_, Ni(BH_4_)_2_, Co(BH_4_)_2_, Hg(BH_4_)_2_, Sc(BH_4_)_3_, Nb(BH_4_)_3_, U(BH_4_)_3_, Ge(BH_4_)_4_, Sn(BH_4_)_4_), while for others that exist as adducts with various stabilizing solvents/ligands (like Cr(BH_4_)_2_), the capacity is reported with respect to the theoretical, anhydrous form [28,29].

Many of the formulated borohydrides from Figure 2 comply with the DOE’s theoretical minimum hydrogen storage capacity (4.5 wt.%), yet very few of them can actually be used in storage materials. However, there are quite a few aspects plaguing metal borohydrides: high dehydrogenation enthalpies, high dehydrogenation temperature onset, slow dehydrogenation/rehydrogenation kinetics, and side-reactions occurring during dehydrogenation leading to boron-loss and thus incomplete/impossible rehydrogenation, but also very high temperature and H_2_ pressures required to rehydrogenate them.

**Figure 3:**
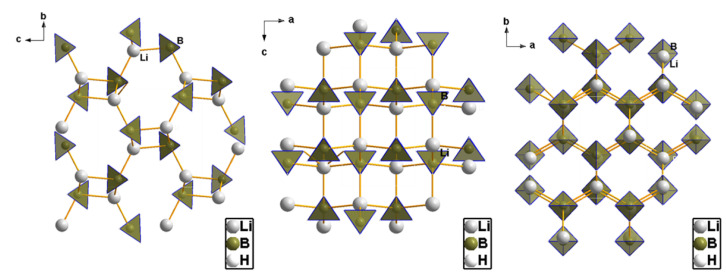
X-ray structure of lithium borohydride, LiBH_4_. Projections along a-axis (**left**), b-axis (**middle**), and c-axis (**right**).

From a structural point of view, borohydrides M(BH_4_)_x_ are ionic/covalent compounds formed by a metal center (M^x+^) that binds accordingly to the negatively charged borohydride tetrahedra BH_4_^−^. The lightest member of the borohydride family is LiBH_4_, depicted in Figure 3 [30,31,32,33,34,35,36,37,38]. Established by synchrotron XRD at RT, LiBH_4_ shows at normal conditions an orthorhombic symmetry (*Pnma*), with the BH_4_ tetrahedra aligned along two orthogonal directions. The [BH_4_]^−^ units are surrounded by four Li^+^ cations, and each Li^+^ by four [BH_4_]^−^.

More important for hydrogenation studies, the B-H bond is of a covalent nature (r_B-H_ = 119 pm), thus considerable energy is required to break that bond (Ε_B-H_ = 389 kJ/mol). It follows that the dehydrogenation enthalpy ΔH_dehydrogenation_ has a large value, an aspect that must be overcome using thermodynamical considerations. First (IA) and second group (IIA) metal borohydrides are not only known, but also commercially available. Various reviews have tackled different areas regarding complex borohydrides and hydrogen, focusing either on current technologies [1,2,6] and conceptual design of storage materials [3,4,5,15], or on various forms of hydrogen carriers [7,8] utilized to eventually switch the aging and depleting conventional fuel to a “green” fuel [9]. Other reviews evaluate the role of nanostructured metal hydride species for hydrogen storage [10,17], and usually focus on the potential presented by light metal borohydrides [10,16,18]. In the latter, sodium borohydride is actually presented as the fuel for the future [18] and judging by the emerging reports dating from 2021–2022 one could argue that this is true. Synthetic strategies and structure characterization [21] have been complemented by in-depth studies on hydrolysis process of light borohydrides [22,24,25,27] or by investigation on the possible intermediates that dehydrogenation could entail [12]. Destabilization strategies employed to tame the rigid behavior of tetrahydroborates during hydrogenation cycles have been reviewed [30,31,32,33,34], and the field of ionic conductivity has been explored with aplomb especially after the discovery of LT(low temperature)–HT(high temperature) phase transition in lithium borohydride [36,37,38]. The current review aims to gather most areas of interest regarding the fascinating chemistry of complex borohydrides and bring them under one umbrella, considering the historical evolution of the field, important milestones, and current and emerging trends. 

## 2. Hydrogen Storage Options: Physical vs. Chemical Storage

**Figure 4:**
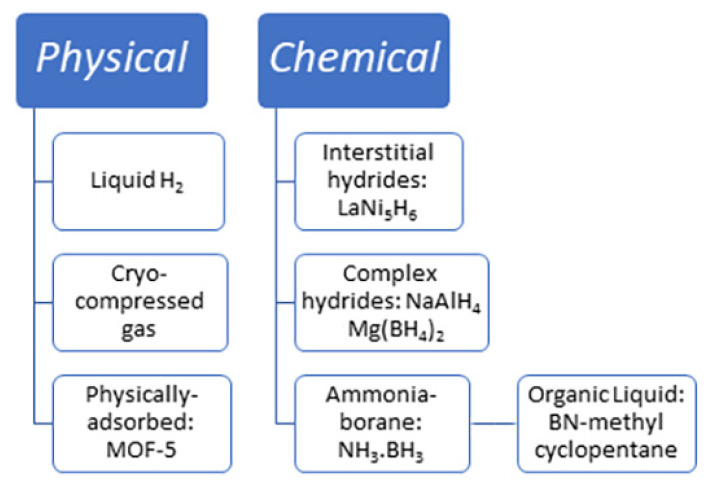
Physical vs. chemical hydrogen storage methods.

Hydrogen storage methods can basically be divided in two groups: physical (liquid H_2_, cryo-compressed and compressed gas, and physically adsorbed as in metal-organic frameworks of MOF-5 type), and chemical storage (organic liquid: BN-methyl cyclopentane; interstitial hydrides: LaNi_5_H_6_; complex hydrides: NaAlH_4_; or in ammonia–borane adducts: NH_3_·BH_3_) (Figure 4).

### 2.1. Physical Storage of Hydrogen

Hydrogen has an atomic weight of 1.0079 g/mol, and is thus the lightest element known. Under normal conditions (standard temperature and pressure), hydrogen is an odorless, nontoxic but combustible gas comprising two hydrogen atoms covalently bonded by a single σ-bond: H_2_. Hydrogen (^1^H, most abundant isotope called protium) has no neutrons, one proton in the nucleus and one electron in the outer shell, and a covalent radius of only 0.315 Å. The common and perhaps the most important physical properties of hydrogen related to its storage applications are the melting point (m.p. = −259.14 °C) and boiling point (b.p. = −252.87 °C), which, coupled with its low density (0.0898 g/L) and high diffusivity, make hydrogen storage a difficult task from an economic and technological point of view. 

Hydrogen has three OS (oxidation states): −1 (hydrides), 0 (molecular dihydrogen), and +1 (typical OS for hydrogen, as found in most compounds, as for instance in hydrocarbons or the ubiquitous H_2_O); it is therefore a versatile element, as it can act as both an oxidizing and a reducing reagent. Its single valence electron makes hydrogen a very reactive element; it is therefore usually found in nature in a variety of inorganic and organic compounds.

Being a fuel and highly flammable (low ignition energy, low explosion limit in air at 4% vol.), hydrogen is regulated through a series of EU safety datasheets: S9 (containers must be kept in well-ventilated areas), S16 (no smoking allowed in its proximity, and ignition sources are forbidden nearby) and S33 (danger due to electrostatic discharge).

Combustion of hydrogen produces the highest gravimetric energy known (33.3–39.4 Wh/g), depending on the state in which water is produced—gaseous water yields the lower value (33.6 Wh/g), while combustion to liquid water produces 39.4 Wh/g:

(3) $$H_{2\left(g\right)}+\frac{1}{2}O_{2\left(g\right)}\;\to \;H_{2}O_{\left(g\right)}\;\;\;\;\;\;\;\;\;\;\;\;\;\;\;\;\;\;\;\Delta H_{1}=-242\frac{\mathrm{kJ}}{\mathrm{mol}};\;33.3\frac{\mathrm{Wh}}{g}$$

(4) $$H_{2\left(g\right)}+\frac{1}{2}O_{2\left(g\right)}\;\to \;H_{2}O_{\left(l\right)}\;\;\;\;\;\;\;\;\;\;\;\;\;\;\;\;\;\;\;\Delta H_{2}=-285\frac{\mathrm{kJ}}{\mathrm{mol}};\;39.4\frac{\mathrm{Wh}}{g}$$

Hydrogen can be stored either as a liquid (cryogenic temperatures, due to low b.p.) or as a gas (at high pressures, in the range 350–700 atm). Physically sorbed hydrogen is also known: adsorption at surface of solids (MOF-5) or absorption (within solids). 

### 2.2. Chemical Storage of Hydrogen

Facing the obvious difficulties of cryogenic temperatures and/or very high pressures, the physical storage of hydrogen has inherent safety issues that have swayed attention of researchers towards chemical storage of hydrogen. Solid-state hydrogen storage can be achieved in metal hydrides, complex metal hydrides, ammonia–borane adducts, or organic liquids (BN-methylcyclopentane) (Figure 4).

Among these, complex hydrides have gained more popularity due to their salt-like nature, high hydrogen storage wt.%, and involvement in a variety of advantageous chemical systems (M(BH_4_)_x_-MH_y_, M(BH_4_)_x_-M(NH_2_)_y_, etc). The general formula of a complex hydride can be written as A_x_D_y_H_z_, with A being usually group IA and IIA elements, and D either boron or aluminum. Complex hydrides contain hydrogen covalently bonded to D, forming the complex anion [D_y_H_z_]^r−^. These compounds have been known for many years, but they were revisited by Bogdanovic and Schwickardy, who discovered that addition of 2 mol % *β*-TiCl_3_ or Ti(OBu)_4_ catalyst to NaAlH_4_ would bring down the kinetic barrier of dehydrogenation to technologically feasible levels for hydrogenation studies. The novel material had a tentative formula NaAlH_3.9_Ti_0.02_Cl_0.06_ and showed a thermogravimetric curve featuring a reduction in the dehydrogenation temperature of 80–85 °C with respect to undoped NaAlH_4_, while proving the reversible character of the prepared sample up to the 100th cycle. However, the dehydrogenation–rehydrogenation rate was still rather low, requiring high temperatures (>150 °C) and pressures (60–150-bar H_2_) (5) [39].

(5) $$NaAlH_{4\;}\Leftrightarrow \frac{1}{3}\;Na_{3}AlH_{6}+\frac{2}{3}\;Al+H_{2}↑\;\Leftrightarrow NaH+Al+\frac{3}{2}\;H_{2}↑$$

This pioneering work opened the door to further investigation of complex hydrides as new motifs for hydrogen storage materials; understanding the role of the catalyst and the exact nature of dehydrogenation and rehydrogenation reactions was the main aim. Other complex hydrides such as Al(BH_4_)_3_ or Mg_2_FeH_6_ have almost double volumetric hydrogen density compared to liquid hydrogen (150 kg/m^3^ vs. 70 kg/m^3^), which stimulated research in this intriguing field of hydrogen storage systems.

## 3. General Synthesis Strategies for Metal Borohydrides M(BH_4_)_x_

There are many known complex metal borohydrides (Figure 2 summarizes most of them). The first known was aluminum borohydride Al(BH_4_)_3_, synthesized in 1939 by Schlesinger et al. by mistake, followed by discovery of NaBH_4_ in 1943 [40]. Out of all known borohydrides, Be(BH_4_)_2_ remains the one providing the highest gravimetric hydrogen content (20.7 wt.%, Figure 2), but it is also very toxic, which limits its usability as hydrogen storage material. While using them as raw materials for hydrogen storage is impractical due to poor reversibility and very high (or low) decomposition temperature, the high amount of hydrogen bound to boron in complex anion [BH_4_]^−^ or [B_2_H_7_]^−^ (the latter is identified only in solution) makes them very interesting starting points for novel energy storage systems.

The general strategies used for metal borohydride synthesis are: solid-state reactions (mechano-chemical), wet chemistry (solvent-assisted), nanoconfined hydrides, and formation of adducts of borohydrides. The mechano-chemical synthesis route is perhaps the most-used and best-known method to obtain metal borohydrides; it is also the only one able to afford mixed-cation borohydrides (like LiZn_2_(BH_4_)_5_) [41]. The main drawback of ball-milling / the mechano-chemical route is the impurity of the resulting borohydride with metal halides, which are by-products of the metathesis reaction [42,43,44,45,46]. By contrast, wet-chemistry methods can yield phase-pure metal borohydrides, and they afford far easier separation procedures. 

### 3.1. Solid-State—Mechanochemical Synthesis

Ball-milling has a series of advantages: it allows for good homogenization of the sample, reduces the size of the particle, and introduces defects which are highly reactive, inclusive in hydrogenation studies [47]. The mechano-chemical route is a highly energetic process carried out usually in a planetary ball mill [47,48,49]. The milling program typically consists in 1–5 min ball-milling followed by a 1–5 min break [50], such that the chemical activation observed is not due to local temperature increase, which is estimated to remain around 60 °C [51,52,53], but rather due to high pressure caused by collisions between ball, powder, and vial which produce material shear stress and deformations [54]. The mechano-chemical approach can increase the reactivity of materials by size-reduction of the particles, which increases their surface area and thereby provides better interaction between particles needed for a complete reaction to occur.

A double substitution reaction (metathesis) occurs when a metal halide (such as MgCl_2_, or more recently MgBr_2_) reacts with NaBH_4_, producing Mg(BH_4_)_2_ in its *β*-phase polymorph:

(6) $$MgBr_{2}+2\;NaBH_{4}\;⟹\beta -Mg{\left(BH_{4}\right)}_{2}+2\;NaBr$$

Using a bromide source rather than chloride yields the expected β-Mg(BH_4_)_2_ faster (6 h), when the reactants are introduced in a near-stoichiometric ratio (MgBr_2_:NaBH_4_ = 1:2 or 1:2.15). The usual workup procedure consists in Soxhlet extraction with Et_2_O, followed by a two-step evaporation under vacuum (150 °C, 24 h and 190 °C, 5 h) [55,56,57,58,59]. The observed reactivity enhancement mirrors the halide reactivity trend, which increases down the 17 group in the order Cl^−^ < Br^−^ < I^−^. 

The seemingly first candidate for hydrogen storage (highest gravimetric hydrogen content, 20.7 wt.%), Be(BH_4_)_2_, was first synthesized by a metathesis reaction, at 145 °C [60]:

(7) $$BeCl_{2}+2\;LiBH_{4}\;⟹Be{\left(BH_{4}\right)}_{2}+2\;LiCl$$

Schlesinger et al. made a great contribution to the development of novel metal borohydrides, after the discovery of Al(BH_4_)_3_ in 1939, and studied the synthesis of borohydrides by metathetical reactions using alkali metal borohydrides [60], alternative syntheses of NaBH_4_ and LiBH_4_ [61], synthesis of NaBH_4_ from NaH and borate esters [62], and synthesis of U(BH_4_)_4_ [63,64]. The metathetical reaction leading to Be(BH_4_)_2_ is not a proper mechano-chemical reaction since it is a solid-state reaction but performed by vigorous shaking of the reagents rather than in a planetary mill [60]. In fact, due to the requirement to continuously remove the volatile (and toxic) Be(BH_4_)_2_ from the system, the reaction cannot be performed in a closed system (like the vial of a planetary mill).

The metathesis is best carried out using LiBH_4_, since it showed considerably faster reactivity towards MCl_x_ than NaBH_4_, having also an added reaction drive due to the formation of stable alkali salt, LiCl [60]:

(8) $$YCl_{3}+3\;LiBH_{4}\;⟹Y{\left(BH_{4}\right)}_{3}+3\;LiCl$$

Synthesis of Y(BH_4_)_3_ starts from yttrium(III) chloride and LiBH_4_, producing in high yield the expected borohydride of Y(III) and LiCl as by-product, in an all-solid metathesis reaction [65,66,67,68,69,70,71,72,73,74,75]. Yan et al. have also tried ball-milling the reagents according to Equation (8) with the aim of obtaining pure Y(BH_4_)_3_, then solvent metathesis of the grinded mixture; however, the obtained yttrium(III) borohydride was only ~85% pure (impurity: LiCl, 15 wt.%) [76]. Moreover, when the stoichiometric mixture YCl_3_: 3 LiBH_4_ was employed for ball-milling at RT or cryo-ball-milling, the reaction mixture showed incomplete conversion to Y(BH_4_)_3_, while YCl_3_: 4 LiBH_4_ yielded almost completely the expected borohydride [67,77,78,79,80]. Usage of LiBH_4_ was compulsory, as Y(BH_4_)_3_ was not obtained when replacing the borohydride source with NaBH_4_ [74]. 

Zr(BH_4_)_4_ can be prepared by a series of solid-state reactions, for instance between NaZrF_5_ + 2 Al(BH_4_)_3_, ZrCl_4_ + 2 Al(BH_4_)_3_, or ZrCl_4_ + 4 LiBH_4_ [77,78,79,80]. 

It has also been suggested that, in the case of mechano-chemical synthesis, the need to evacuate the volatile borohydride species (Be(BH_4_)_2_ or Al(BH_4_)_3_ being prime examples) advocates for an equilibrium reaction that is shifted towards products with removal of the volatile product, in line with Le Chatelier’s principle [81,82]. Al(BH_4_)_3_ has a theoretical hydrogen storage capacity of 16.8 wt.%, but is a liquid at RT (mp = −64 °C) due to weak intermolecular forces between its molecules; it is fairly unstable and begins to decompose at ~40 °C, which is too low for use in a hydrogen production mobile tank. Its crystal structure presents [BH_4_^−^] units that are covalently bonded by two H-bridges to the Al^3+^ center. A ball-milling reaction of AlCl_3_ with NaBH_4_ in a stoichiometric molar ratio results in good yields of Al(BH_4_)_3_ [60].

(9) $$AlCl_{3}+3\;NaBH_{4}\;⟹Al{\left(BH_{4}\right)}_{3}+3\;NaCl$$

The solid-state Al(BH_4_)_3_ has two polymorphs (transition temperature −93 to −78 °C) [83,84,85,86,87,88]. Due to its liquid nature, toxicity, and moisture and air sensitivity, Al(BH_4_)_3_ rather serves as a starting point for synthesis of other, more stable borohydrides, and it is not currently the focus of research on novel energy storage materials because of these limitations. 

**Figure 5:**
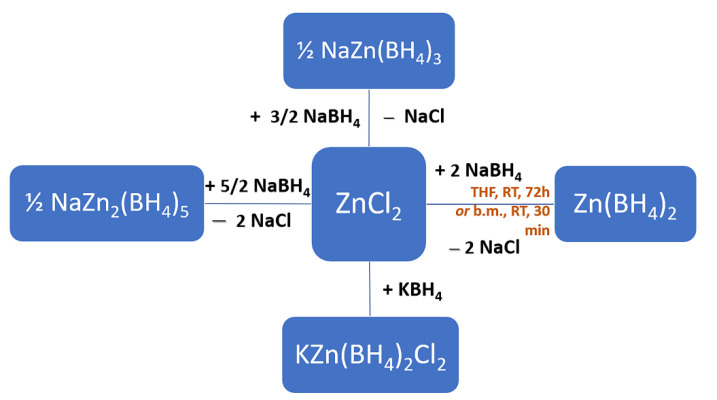
Complexity of ball-milling process, exemplified for synthesis of Zn(BH_4_)_2_ from ZnCl_2_ and MBH_4_ (M = Na, K) under various ratios of starting materials (1:1, 1:1.5, 1:2, and 1:2.5); THF-tetrahydrofuran, RT-room temperature, b.m - ball milling.

Ball-milling is also the only way to produce mixed-cation or mixed-anion borohydrides, with reaction conditions (milling time, temperature, pressure) depending ultimately on the metal electronegativity and d-electron configuration in the case of transition metal systems. For instance, mixing ZnCl_2_ and KBH_4_ in a 1:1 ratio produces a chloro-borohydride of K^+^ and Zn^2+^. The complexity of the ball-milling process and its high dependence on reagent molar ratio is depicted below, showing a family of products all starting from ZnCl_2_ and a metal alkali borohydride source (Figure 5) [41]. 

Besides the formation of LiCl (or NaCl) as by-products, the metathetic route has another drawback: some amounts of this salt can dissolve in LiBH_4_, yielding a solid solution of approximate formula Li(BH_4_)_1-p_Cl_p_ (p is an integer number, 0 < *p* < 1), making further purification even more difficult [89]. 

Synthesis of borohydrides can also be accomplished from starting elements as raw materials, but under extreme conditions caused by the very low reactivity of boron, as shown by Friedrichs et al. (700 °C, 150 bar) [90,91]:

(10) $$Li+B+2\;H_{2}\;⟹LiBH_{4}$$

Other high-pressure reaction routes starting from solid metal hydride or metal boride have been investigated. For instance, reacting metal hydride MH_x_ with MgB_2_ and hydrogen H_2_ leads to corresponding M(BH_4_)_x_ and MgH_2_ [92,93].

(11) $$MH_{x}+\frac{x}{2}\;MgB_{2}+2x\;H_{2}\;⟹M{\left(BH_{4}\right)}_{x}+\frac{x}{2}\;MgH_{2}$$

 for x=1, M=Li, Na; for x=2, M=Ca

Regeneration of Ca(BH_4_)_2_ during hydrogenation studies was shown to proceed at 350 °C and 150-bar H_2_, with a faster dehydrogenation/rehydrogenation kinetics when a Ti-catalyst was employed [94]. 

Ca(BH_4_)_2_ can be obtained in good yield (60%) by rehydrogenation of calcium hexaboride CaB_6_ and CaH_2_ under harsh conditions (690–700-bar H_2_, 400–460 °C) [95]:

(12) $$2\;CaH_{2}+CaB_{6}+10\;H_{2}\;⟺3\;\alpha -Ca{\left(BH_{4}\right)}_{2}$$

Interesting to note in Equation (12) is the formation of *α*-Ca(BH_4_)_2_ at temperatures above 400 °C; typically, when synthesized at lower temperature, *α*-Ca(BH_4_)_2_ undergoes an irreversible phase transformation to *β*-Ca(BH_4_)_2_ when heated above 180 °C; this synthesis route thus has a main advantage in affording the *α-*phase at high temperatures (Equation (32)). The overall hydrogenation capacity of Ca(BH_4_)_2_ based on Equation (12) is 9.6 wt.%. Formation of CaB_6_ instead of the unreactive B or high boranes like CaB_12_H_12_ in the dehydrogenation process confers to the reaction a real potential for reversibility [96,97,98,99]. The formation of Ca(BH_4_)_2_ can also take place in modest yields (~20%) by ball-milling at RT for 24 h and under 140-bar H_2_, when using TiF_3_ as catalyst; in fact, many rehydrogenation reactions benefit from using Ti-based catalysts [100]. Ball-milling under Ar backpressure (1 atm) between CaCl_2_ and MBH_4_ (M = Li, Na) was reported to also produce Ca(BH_4_)_2_, albeit made impure with chloride by-products (Equation (13)) [101].

(13) $$CaCl_{2}+2MBH_{4}\overset{Ar,\;1\;atm}{\Rightarrow }\alpha -Ca{\left(BH_{4}\right)}_{2}+2\;MCl\;;M=Li,\;Na$$

Reaction between sodium hydride NaH and boric oxide have produced sodium borohydride in good yield (60%) after ball-milling at 330–350 °C for 20–48 h [62]:

(14) $$4\;NaH+2\;B_{2}O_{3}\;\Rightarrow NaBH_{4}+3\;NaBO_{2}$$

In a similar fashion, but using methyl borate B(OCH_3_)_3_ instead of boric oxide, LiBH_4_ was obtained with an overall yield of about 70% [62]:

(15) $$4\;LiH+B{\left(OCH_{3}\right)}_{3}\;\Rightarrow LiBH_{4}+3\;LiOCH_{3}$$

However, the economical aspect of using mechano-chemical synthesis may tip the balance, because large-scale facilities for grinding are known and operable, so that cost-effectiveness is insured for the preparation of light complex hydrides via the ball-milling technique.

### 3.2. Wet Chemistry—Solvent-Assisted Synthesis

The first borohydride, Al(BH_4_)_3_, was synthesized by Schlesinger by accident in 1939 [40] (Equation (16)). He was hoping to obtain AlH_3_ by mixing Al(CH_3_)_3_ and B_2_H_6_, when the following reaction actually occurred, which has further been expanded to the synthesis of Be(BH_4_)_2_ (Equation (17)) and LiBH_4_ (using an alkyl-lithium in the latter case). LiBH_4_ is the second most used borohydride today, but it was first synthesized also in the 1940s by Schlesinger et al. according to Equation (18) [102].

(16) $$Al{\left(CH_{3}\right)}_{3}+2\;B_{2}H_{6}\;⟹Al{\left(BH_{4}\right)}_{3}+3\;{\left(CH_{3}\right)}_{3}B$$

(17) $$3\;Be{\left(CH_{3}\right)}_{2}+4\;B_{2}H_{6}\;⟹3\;Be{\left(BH_{4}\right)}_{2}+2\;{\left(CH_{3}\right)}_{3}B$$

(18) $$3\;LiC_{2}H_{5}+2\;B_{2}H_{6}\;⟹3\;LiBH_{4}+{\left(C_{2}H_{5}\right)}_{3}B$$

At present, the usual procedure for the synthesis of M(BH_4_)_x_ follows the general reactivity scheme (19) and (20), and it can be successfully applied for divalent borohydride synthesis (M = Be, Mg, Ca, Sr, Ba) or trivalent borohydrides (M = Al):

(19) $$LiX+NaBH_{4}⟹\;LiBH_{4}+\;NaX\;\;\;\;\;\;\;\;\left(X=Cl,\;Br,\;I\right)$$

(20) $$MCl_{x}+x\;LiBH_{4}⟹M{\left(BH_{4}\right)}_{x}+x\;LiCl$$

Al(BH_4_)_3_ was obtained by Kollonitsch and Fuchs and reported back in 1955, by reacting AlCl_3_ with Ca(BH_4_)_2_ in a 2:3 molar ratio [103].

(21) $$2AlCl_{3}+3Ca{\left(BH_{4}\right)}_{2}⟹2Al{\left(BH_{4}\right)}_{3}+3CuCl_{2}$$

Synthesis of LiBH_4_ according to Equation (19) takes place in NaH-dried isopropylamine *^i^*PrNH_2_ [60,104], and usage of toxic diborane B_2_H_6_ is now avoided as in its first synthesis by Schlesinger and Brown [102]. The metathesis reactions (19) and (20) are well-documented, and proceed with a reasonable rate for LiBH_4_ compared to slower alkali borohydride starting materials (i.e., NaBH_4_, and ever slower with KBH_4_) [60], and afford the complex borohydride M(BH_4_)_x_ in near-quantitative yield. NaBH_4_ remains the most wide-spread borohydride in terms of synthesis methods and uses, including but not limited to organic synthesis, reduction reactions, and metathesis [105]. 

Mg(BH_4_)_2_ could be synthesized according to Equation (20), using diethyl ether Et_2_O as solvent for the LiBH_4_, but the product obtained contains impurities of Li and Cl^−^ [106,107,108]. At modest temperatures (34–60 *°*C), reaction (22) yields some Mg(BH_4_)_2_ (overall yield: 0–11.5%); different conditions must therefore be used if NaBH_4_ is used as borohydride source [107,108]. Even when conducted in recommended solvent *^i^*PrNH_2_, the final mixture could not surpass 40 wt.% of Mg(BH_4_)_2_, with the impurities being mainly represented by unreacted NaBH_4_; this aspect could be proof of an equilibrium reaction being reached under those specific reaction conditions, but it could also originate from poor solubility of the intermediate magnesium amine complex formed in the reaction mixture [106]. Phase-pure β-Mg(BH_4_)_2_ was obtained by replacing highly reactive LiBH_4_ with NaBH_4_, using a combined mechano-chemical (2 h) double exchange reaction (60 h, refluxing Et_2_O solvent), followed by drying at 240 °C [109].

(22) $$MgCl_{2}+2\;NaBH_{4}\;⟹Mg{\left(BH_{4}\right)}_{2}+2\;NaCl$$

Organometallic compounds can also be used to synthesize metal borohydrides; reaction of n-butyl-magnesium with Al(BH_4_)_3_ in non-coordinating solvent (toluene) yields a solvent-free Mg(BH_4_)_2_ in very good yield (85%) (Equation (23)) [110].

(23) $$3\;Mg{\left(C_{4}H_{9}\right)}_{2}+2\;Al\;{\left(BH_{4}\right)}_{3}\;⟹3\;Mg{\left(BH_{4}\right)}_{2}+2\;Al\;{\left(C_{4}H_{9}\right)}_{3}$$

Reaction of CaH_2_ or Ca(OC_2_H_5_)_2_ with diborane B_2_H_6_ in THF also affords the adduct of Ca(BH_4_)_2_ in good yield [111,112,113,114,115,116,117]. The reaction of ball-milled activated CaH_2_ (1 h, under Ar, 600 rpm) and triethylamine borazane complex Et_3_N·BH_3_ produces Ca(BH_4_)_2_ in 85% yield (unreacted CaH_2_ as main impurity). The reaction is carried out at 140 °C for 5–6 h and the subsequent workup consists in *n-hexane* washing, filtering, and drying at 200 °C for 12 h [118]. 

Zn(BH_4_)_2_ could also be prepared by wet-chemistry at room temperature (RT), using tetrahydrofuran (THF) as solvent and ZnCl_2_ and NaBH_4_ as starting materials (Figure 5) [119,120,121,122]. With a hydrogen storage capacity of 8.5 wt.%, Zn(BH_4_)_2_ starts to decompose at ~85 °C and eliminates up to 140 °C hydrogen and diborane B_2_H_6_, making it for the time being too unstable for vehicular applications. On the other hand, formation of toxic B_2_H_6(g)_ should urge researchers to look for alternative decomposition pathways that avoid diborane as by-product, since current dehydrogenation will not be reversible in the absence of the boron source [119,120,121,122]. 

Potassium borohydride KBH_4_ (also RbBH_4_ and CsBH_4_) could be synthesized by a precipitation reaction of NaBH_4_ with a concentrated solution of a strong base, KOH; the reaction could be carried out in aqueous or CH_3_OH media, with an overall yield of 75.5% (Equation (24)). The reagents are independently dissolved in MeOH, cooled, and then mixed, when the borohydride of interest precipitates and is obtained in pure form after a subsequent vacuum drying step [123,124].

(24) $$NaBH_{4}+MOH\;⟹MBH_{4}+NaOH\;\;\;\;\;\left(M=K,\;Rb,\;Cs\right)$$

LiBH_4_ can also be produced by reacting LiH with diborane at mild temperatures (120 °C) [125]. However, no reaction takes place between LiH and CaB_2_; Ca(BH_4_)_2_ therefore cannot be obtained via this route (Equation (25)) [92].

(25) $$2\;LiH+B_{2}H_{6}\;\;⟹2\;LiBH_{4}$$

A similar reaction between the metal hydride MH_x_ (M = Li, Mg, Ca) and B_2_H_6_ in a solid–gas mechanochemical process was proposed by Friedrichs et al., who obtained borohydride products in good-to-high yield, but containing some impurities (unreacted starting metal hydride and Fe-Ni phases from vial abrasion during milling): LiBH_4_ (η = 94%), Mg(BH_4_)_2_ (η = 91%) and Ca(BH_4_)_2_ (η = 73%). Diborane B_2_H_6_ was also proposed as a key intermediate in hydrogenation studies under moderate conditions [93].

Neutron diffraction studies have been facilitated by a hydrogen-deuterium H-D exchange reaction taking place at increasing temperatures for Li, Na, and K (200 °C, 350 °C, and 500 °C, respectively) (Equation (26)) [126].

(26) $$\;MBH_{4}+2D_{2}\;\;⟹\;MBD_{4}+2H_{2}\;\;\;\;\;\;\;\;\;\;\;\left(M=Li,\;Na,\;K\right)$$

### 3.3. Nanoconfined Hydrides

The ball-milling technique is usually the go-to process for obtaining nanosized powders of metal hydrides, to sizes often less than 100 nm [127]. Ball-milling implies milling in a vial using balls of special alloys; however, the milling process could induce impurities in the sample from the materials of the vial and balls. However, these nanoparticles could also sinter into larger particles during hydrogenation–dehydrogenation cycles, which negatively impacts gravimetric hydrogen uptake and release [128,129,130,131]. Using specially engineered nano-scaffolds, complex hydrides can be obtained in the nanometer range, far below the average sizes afforded by the mechano-chemical approach.

Among nanoconfinement methods, the most widely used are the direct synthesis of nanoconfined complex metal (boro)hydride, and infiltration methods (melt-infiltration and wet-infiltration) [132]. 

**Figure 6:**
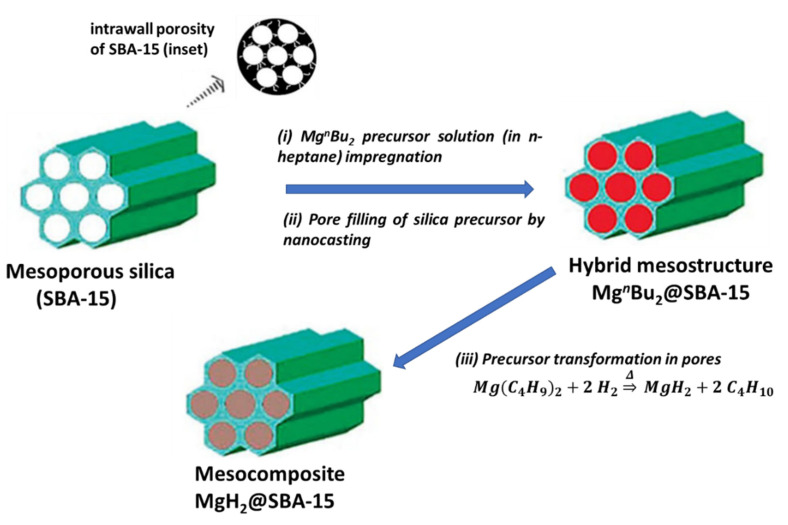
Soft nanocasting showing infiltration of Mg*^n^*Bu_2_ solution into SBA-15 mesoporous silica template to yield finally porous MgH_2_ nanoconfined inside SBA-15 pores (Dp~7.5 nm).

Nanoconfinement of metal hydrides synthesized in situ has been extensively studied for magnesium hydrides in carbon aerogels produced by the resorcinol–formaldehyde reaction (RF-CA), using a 1 M solution of Mg(^n^C_4_H_9_)_2_ in n-heptane [133,134]. Nielsen et al. synthesized by this method MgH_2_@RF-CA of pore sizes 7 and 22 nm [133]. This is a typical case of soft-nanocasting, as the initial porous support is not removed from the final composite material (Figure 6). 

The excess n-butyl magnesium can be removed manually after the reaction from the surface of RF-CA (Equation (27)).

(27) $$Mg{\left(C_{4}H_{9}\right)}_{2}+2\;H_{2}\;\overset{\Delta }{\Rightarrow }\;MgH_{2}+2\;C_{4}H_{10}$$

Melt infiltration requires a very inert scaffold, with a melting point higher than that of the respective metal hydride used. The driving force of this method is the lower interfacial energy metal hydride/scaffold, as reviewed by de Gennes [135,136]. This method has the advantage of completely removing the necessity of a solvent, therefore avoiding post-synthesis steps of the hydride@scaffold composite. In addition, the melting point of the hydride must not coincide with the onset of decomposition of the said material. However, sodium tetrahydroaluminate NaAlH_4_ (mp = 183 °C) has been utilized for melt infiltration, despite decomposition onset into Al and Na_3_AlH_6_ upon melting [137]. 

The decomposition of NaAlH_4_ must be considered a partial equilibrium (Equations (28) and (29)), because when working around the borohydride melting point and using high H_2_ pressure (160–190 bar), the decomposition is avoided and melt infiltration has been successful [137,138,139,140,141,142].

(28) $$3\;NaAlH_{4}\;\;\overset{\Delta }{\Leftrightarrow }\;Na_{3}AlH_{6}+2\;Al+3\;H_{2}$$

(29) $$\;Na_{3}AlH_{6}\;\;\overset{\Delta }{\Leftrightarrow }3\;NaH+Al+\frac{3}{2}\;H_{2}$$

Solvent infiltration requires a suitable solvent that affords complete metal hydride dissolution in order to be successful. A series of physical properties of metal borohydrides (including knowledge of select solubility data) are a prerequisite, as limited solubility in commonly used solvents is the main limitation of this method. Sometimes even weakly coordinating solvents could be used, but these solvents should be removed under moderate conditions (T, p). Following the rather poor solubility of complex metal hydrides, consequent steps of impregnation–evaporation may be required to reach full (or the desired degree of) scaffold pore filling [132]. This technique—incipient wetness method—can be advantageous as it avoids crystallization of active hydride species outside of mesopores; complex metal borohydrides like Ca(BH_4_)_2_ have been infiltrated in chemically activated micro-mesoporous carbons of very high surface area to produce composites exhibiting reversible hydrogen storage under more modest conditions [143].

Various scaffolds can be used; inert, mesoporous silica of 2D-ordered type (SBA-15) could serve such a purpose. Computational studies have shown that molecules of NH_x_BH_x_ type (x = 1–4) display thermodynamically accessible dehydrogenation steps of less than 40 kJ/mol, and relatively low dehydrogenation temperature onset (100–150 °C); they can thus be considered candidates for hydrogen storage reactions [144]. When a 5.4 M solution of ammonia–borane NH_3_.BH_3_ in CH_3_OH infiltrated the ~7.5 nm pores of SBA-15 silica, a total loading of roughly 50 wt.% NH_3_BH_3_@SBA-15 was obtained after solvent evaporation [145]. A clear advantage of solvent-mediated impregnation is that the experimental procedure requires less energy, since it can be carried out even at room temperature. 

### 3.4. Derivatives—Formation of Adducts of M(BH_4_)_x_

Solvent metathesis reactions often lead to the formation of desired borohydrides as solvates: M(BH_4_)_x_·*p* Et_2_O (p is an integer number), as is the actual case for M = Mg and Y. Both Mg(BH_4_)_2_·*p* Et_2_O [106,107,108] and Y(BH_4_)_3_·*p* Et_2_O [76] could be obtained as pure, unsolvated solids after heating at 150 °C, as shown by IR and TG data (Equation (30)).

(30) $$M{\left(BH_{4}\right)}_{x}\cdot p\;Et_{2}O\;\overset{150\;{}^{\circ}C}{\Rightarrow }M{\left(BH_{4}\right)}_{x}+p\;Et_{2}O\;\;\;\;\;\;\left(M=Mg,\;Y\right)$$

Other borohydrides are obtained as tetrahydrofuran adducts, such as Ca(BH_4_)_2_·*p* THF, which is also commercially available. Heating this adduct at 160 °C for 1 h leads to phase-pure, *α*-Ca(BH_4_)_2_ (Equation (31)) [146,147,148,149,150,151]. Careful control of the temperature profile is advised, as from 180 °C the conversion to *β*-Ca(BH_4_)_2_ occurs (Equation (32)).

(31) $$Ca{\left(BH_{4}\right)}_{2}\cdot p\;THF\;\overset{\begin{array}{c} 160\;{}^{\circ}C,\\ \;1h \end{array}}{\Rightarrow }\alpha -Ca{\left(BH_{4}\right)}_{2}+p\;THF$$

(32) $$\alpha -Ca{\left(BH_{4}\right)}_{2}\;\overset{180-200\;{}^{\circ}C\;}{\Rightarrow }\;\beta -Ca{\left(BH_{4}\right)}_{2}$$

As is typical with organometallic synthesis, purification of metal borohydrides can be achieved using recrystallization from a concentrated solution (usually the synthesis solvent: *^i^*PrNH_2_, MeOH or even H_2_O). NaBH_4_ could be recrystallized from *^i^*PrNH_2_ [152] or H_2_O [153]. In the latter case, single crystals could be obtained corresponding to dihydrate sodium borohydride, NaBH_4_·2H_2_O. 

Triethylamine (Et_3_N)-solvated metal borohydrides: NaBH_4_ or partially deuterated NaBD_3_H [154,155], Mg(BH_4_)_2_ [156], and Ca(BH_4_)_2_ [157], could be obtained by reacting the corresponding premilled (and thus highly reactive) metal hydride with an excess of aminoborane (Equations (33) and (34)).

(33) $$NaH+Et_{3}N\cdot BD_{3}\;\;\overset{\;}{\Rightarrow }\;NaBD_{3}H+Et_{3}N\;$$

(34) $$MH_{2}+2\;Et_{3}N\cdot BH_{3}\;\;\overset{\;}{\Rightarrow }\;M{\left(BH_{4}\right)}_{2}\;\cdot \;2\;Et_{3}N\;\overset{\Delta \;}{\Rightarrow }\;\alpha -M{\left(BH_{4}\right)}_{2}+2\;Et_{3}N\;\;\left(M=Mg,\;Ca\right)$$

Dimethyl sulfide S(CH_3_)_2_-stabilized magnesium borohydride can also be obtained, provided that a large excess of the tioborane is used. Solvent-free Mg(BH_4_)_2_ can be obtained by working up the reaction mixture at low pressures (10^−1^–10^−5^ mbar, for 19 h in total), a step which removes coordinating solvent S(CH_3_)_2_ (Equations (35) and (36)) [110].

(35) $$3\;Mg{\left(C_{4}H_{9}\right)}_{2}+8\;BH_{3}\cdot S{\left(CH_{3}\right)}_{2}\;⟹3\;Mg{\left(BH_{4}\right)}_{2}\cdot 2\;S{\left(CH_{3}\right)}_{2}+2\;B{\left(C_{4}H_{9}\right)}_{3}\cdot S{\left(CH_{3}\right)}_{2}$$

(36) $$Mg{\left(BH_{4}\right)}_{2}\cdot 2\;S{\left(CH_{3}\right)}_{2}\overset{\Delta \;}{\Rightarrow }\;\alpha -M{\left(BH_{4}\right)}_{2}+2\;S{\left(CH_{3}\right)}_{2}$$

Desolvation from a dimethylsulfide borane complex solvate led instead to a new, nanoporous polymorph, γ-Mg(BH_4_)_2_ (Equation (37)) [158].

(37) $$S{\left(CH_{3}\right)}_{2}\cdot BH_{3}\overset{\;Mg{\left(C_{4}H_{9}\right)}_{2}\;}{\Rightarrow }Mg{\left(BH_{4}\right)}_{2}\cdot \frac{1}{2}S{\left(CH_{3}\right)}_{2}\overset{\;vacuum;\;RT\to 80\;{}^{\circ}C\;}{\Rightarrow }\;\gamma -Mg{\left(BH_{4}\right)}_{2}$$

The structural variety of polymorphs obtained in the case of magnesium borohydride (six in total) (Equations (35)–(37)) suggests that novel, nanoporous metal hydrides could be potentially accessible via this desolvation route.

**Figure 7:**
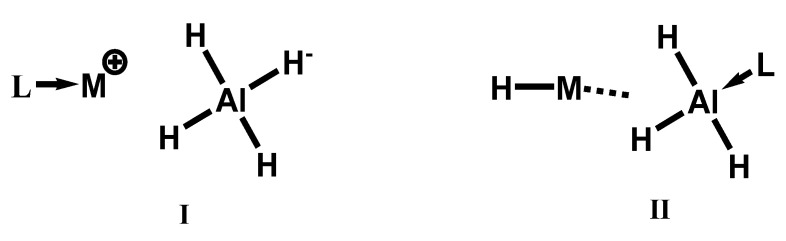
Two formulations (**I** [102] and **II** [159]) of possible solvate structures for ligand-coordinated metal alanates of select alkali metals (M: Li, Na).

Thermal analysis coupled with IR spectroscopy data has pointed out two possible formulations of solvated complexes of closely related tetrahydroaluminates, when coordinated to a tertiary amine “L” (Figure 7). If a weak coordination is expected, then structural formula **I** is favorable, and it should resemble that of an uncoordinated complex metal hydride, as pointed out by Schlesinger and Brown. The complex should exhibit high thermal stability—which is actually the case for **I**: L → M^+^ [AlH_4_]^−^, due to the increased size of the cation and the reduced distortion of the AlH_4_^−^ tetrahedron by alkali metal interaction [102]. By contrast, formula **II**: H-M…AlH_3_L is expected to have a lower thermal stability, resulting in a complex mixture of products upon decomposition due to the complex bond amine-alanate R_3_N: →AlH_3_ [159]. 

Full decomposition of solvate LiBH_4_·L could proceed through either pathway (38) or (39) as evidenced by the ambiguous DTA-TGA curve, with both leading to loss of coordinating molecule L:

(38) $$LiAlH_{4}\cdot L\;\;\overset{\;}{\Rightarrow }\;LiH+Al+\frac{3}{2}H_{2}+L$$

(39) $$3\;LiAlH_{4}\cdot L\;\;\overset{\;}{\Rightarrow }\;\;Li_{3}AlH_{6}+2\;Al+6\;H_{2}+3\;L$$

Mg(BH_4_)_2_·THF adducts decompose violently [160] due to the formation of alkoxy species by the THF ring-opening reaction, as is the case with aluminum hydride-THF adducts [161]. However, Ca(BH_4_)_2_·THF can be desolvated with relative ease, without such strong exothermic effects, as evidenced by Equation (31) [146,147,148,149,150,151,162]. 

A metathesis reaction is currently the preferred method of synthesis for metal borohydrides because they can be obtained in pure form. There are recent reports expanding the recognized reducing character of metal borohydrides. For instance, Ca(BH_4_)_2_ can be successfully used in advanced organic synthesis, such as polymerization of ε-caprolactine and L-lactide [163].

## 4. Structural Considerations of M(BH_4_)_x_

There is an intricate connection between structure and solid-state chemistry related to borohydride materials. Among most important physical properties affected by lattice structure are the thermal stability and the accessible decomposition pathways.

### 4.1. Framework and Crystal Structure

Metal borohydrides are typically ionic compounds, but covalent borohydrides are also known. Table 1 summarizes some of the novel metal borohydrides and mixed-cation borohydrides and their structural architecture; many present multiple polymorphism (LT, HT, metastable). 

**Figure 8:**
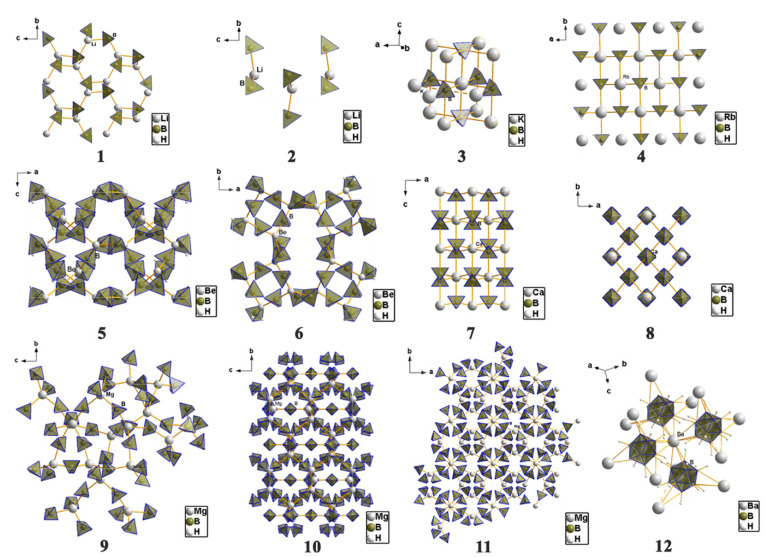
Structural diversity exhibited by known and characterized main group (alkali and alkali-earth) metal borohydrides. Crystal system for various borohydrides depicted as projections along “a” crystallographic axis. **1**—LiBH_4_ (*α*-, LT polymorph, a-axis view); **2**—LiBH_4_ (*α*-, HT polymorph, a-axis view); **3**—KBH_4_; **4**—RbBH_4_; **5**—Be(BH_4_)_2_ (b-axis view); **6**—Be(BH_4_)_2_ (c-axis view); **7**—*α*-Ca(BH_4_)_2_ (b-axis view); **8**—*α*-Ca(BH_4_)_2_ (c-axis view); **9**—*α*-Mg(BH_4_)_2_ (a-axis view); **10**—*β-*Mg(BH_4_)_2_ (a-axis view); **11**—*γ*-Mg(BH_4_)_2_ (c-axis view); **12**—BaB_12_H_12_.

The structural diversity of metal borohydrides is stunning, and a glimpse of such diversity is provided in Figure 8, with a few representative examples. For instance, CsSr(BH_4_)_3_ exhibits a structure similar to that of perovskite, which on the one hand explains its high stability with a decomposition onset at 398 °C [185]. Potassium octahydridotriborate *β*-KB_3_H_8_, on the other hand, exhibits discrete units implying a high mobility of boron tetrahedra during chemical transformations [186].

Perhaps one of the most studied metal borohydrides of the main groups, LiBH_4_, (**1** in Figure 8) crystallizes in the orthorhombic *Pnma* space group, with cell dimensions *a* = 7.1785 Å, *b* = 4.4368 Å, *c* = 6.8032 Å, and *V* = 216.685(3) Å^3^ at 20 °C. While early diffraction data of lower resolution suggesting strong distortion of the BH_4_ tetrahedra, more recent determinations show that B-H bonds and H-B-H angles practically describe perfect tetrahedrons [30,31,32,33,34,35,36,37,38]. Each Li^+^ cation coordinates four [BH_4_]^−^ tetrahedra, and each [BH_4_]^−^ tetrahedron coordinates four Li^+^ cations. The LT polymorph of LiBH_4_ shows the Li^+^ cation in four-coordinate tetrahedral sites between anionic layers of [BH_4_^−^] that are hexagonally stacked. When heated to ~110 °C, the LT-LiBH_4_ (o-LiBH_4_) undergoes a phase transition to a hexagonal polymorph, HT-LiBH_4_ (h-LiBH_4_), which crystallizes in hexagonal space group P6_3_mc resembling the wurtzite (ZnS) structure, with the following unit cell parameters: *a* = 4.2763 Å, *c* = 6.9484 Å, and *V* = 110.041(4) Å^3^ at 135 °C (**2**—in Figure 8). The *o*-LiBH_4_ to *h*-LiBH_4_ polymorphic transformation taking place at ~110 °C can be seen as a contraction along orthorhombic axis *a* (hexagonal axis *c*) and an expansion of the [b*c] orthorhombic plane, and it is accompanied by a slight endothermic enthalpy of transition (4.18 J/mol). 

KBH_4_ (**3** in Figure 8) in its *β*-polymorph, crystallizes in the tetragonal *P4_2_/nmc* space group. The 3D structure shows a 12-coordinate geometry around the alkali metal, in an equivalent H-surrounding. The K-H bond lengths average 2.815 Å, while the B-H bonds in the [BH_4_]^−^ unit measure 1.23 Å. The structure of *β-*KBH_4_ bears structural similarities to tetragonal NaBH_4_, but it is refined to a higher symmetry group *P4_2_/nmc.* As the size of the cation increases in the alkali group (from Li to Rb), the cubic MBH_4_ (M = alkali metal) unit cell expands and the [BH_4_]…[BH_4_] repulsions are reduced, such that the cubic-to-tetragonal transition temperature is reduced in heavier alkali metal borohydrides. 

RbBH_4_ (**4** in Figure 8) crystallizes in the cubic *Fm*$\bar{3}$*m* space group (a = 6.9633 Å at 0.5 GPa). The Rb^+^ cations and BH_4_^−^ anions have a NaCl structural prototype and this room-temperature polymorph is isostructural to NaBH_4_, with the BH_4_ groups orientationally disordered over the two positions around the inversion center, similar to the sodium tetrahydroborate case. 

Be(BH_4_)_2_ (**5** in Figure 8) presents only one structurally characterized form which crystallizes in the tetragonal *I4_1_/cd* space group. It contains helical polymeric chains with the independent Be^2+^ coordinated in a trigonal-planar geometry to two bridging [BH_4_] tetrahedra via face coordination (Be-B bond distance 2.00 Å) and one terminal [BH_4_] tetrahedron via the tetrahedral edge (Be-B bond distance 1.92 A). This originates presumably from the repulsive [BH_4_]…[BH_4_] forces due to very short H…H distances (ranging from 2.24 Å to 2.31 Å). The bridging [BH_4_] group shows a linear coordination to the two Be^2+^ cations (Be-B-Be angle close to 180°), leading to a 1D polymeric structure.

*α*-Ca(BH_4_)_2_ (**7** in Figure 8) can be obtained by desolvation of the THF adduct, and crystallizes in the orthorhombic *Fddd* space group, being one of the four possible polymorphs (*α*, *α’*, *β* and *γ*) and bearing structural similarities to TiO_2_ polymorphs [187,188]. The orthorhombic structure has been further amended to better be represented by a lower symmetry (*F2dd* or *Fdd2*). All four polymorphs show Ca^2+^ octahedrally coordinated to six equivalent [BH_4_] tetrahedra, with the Ca-B bond distances falling in the range 2.82–2.97 Å. The divalent calcium cations form in *α*-Ca(BH_4_)_2_ a close-packed, diamond-type structure where the tetrahydroborate groups exhibit T-shape coordination [188]. 

Three polymorphs of Mg(BH_4_)_2_ are depicted (**9**: *α*-, **10**: *β*-, **11**: *γ*-), showing increased structural complexity, but also polymeric properties [106,158,171,172,173,189,190,191,192,193,194,195]. The *α*-Mg(BH_4_)_2_ represents the most stable polymorph and its structure determination was a result of combined synchrotron (most information data) and neutron powder diffraction data (orientation of BH_4_ tetrahedra). Single crystal diffraction data collected at 100 K established the space group to be the hexagonal *P6_1_22*, containing three symmetry-independent Mg^2+^ ions and six independent [BH_4_] units. In this novel 3D arrangement, the Mg-Mg distances lie in the range 4.6–4.9 Å, while the Mg^2+^ center is tetrahedrally coordinating four [BH_4_^−^] tetrahedra. The Mg-B bond lengths are in the range 2.432(a)–2.434(4) Å, and there is a dispersity of B-Mg-B angles of 92.1(2)–108.80(13)° accounting for a distorted tetrahedral coordination around the metal center. The porous *γ-*Mg(BH_4_)_2_ has a cubic lattice with inherited porous nature (33% open space), high surface area (in excess of 1100 m^2^/g as deduced from N_2_ sorption isotherms by BET method) and a partially covalent framework. The stability of Mg(BH_4_)_2_ can be related to the Mg-BH_4_-Mg linear units, with BH_4_ acting as directional ligands, and stable MgH_8_ polyhedra. This complex structural behavior resembles that of coordination polymers like MOFs. The *γ*-polymorph is particularly interesting due to large empty spaces available for gas entrapment [158]. 

One of the main dehydrogenation borohydride species found during hydrogen desorption studies is the [B_12_H_12_]^2−^ anion, as found for instance in BaB_12_H_12_ (**12** in Figure 8). These dodecahydro-*closo*-dodecaborate anions [B_12_H_12_]^2−^ have a role in the poor recyclability of metal tetrahydroborates from hydrogenation studies; their formation and possible side-reactions are therefore of clear importance. Moreover, it has been hypothesized that these species appear amorphous in XRD diffraction data, and only show short-range ordering. BaB_12_H_12_ crystallizes in the *P31c* space group (trigonal symmetry) of wurtzite structural type, and shows Ba^2+^ cation surrounded by four [B_12_H_12_]^2−^ anions. Each *closo*-borate [B_12_H_12_]^2−^ anion is tetrahedrally surrounded by four Ba^2+^ cations (mutual tetrahedral arrangement of cations and anions), with distances from the center of the anion to the Ba^2+^ of 4.40 and 4.54 Å at 295 K. Since each of the four [B_12_H_12_]^2−^ anions is facing the Ba^2+^ metal center with three H atoms, the cation is surrounded overall by twelve H atoms (Ba–H bond distances range from 2.77–3.02 Å). The rich boron–hydrogen speciation, open channels, and convenient molecular size (5.8 Å) have made *closo*-borate salts very interesting candidates for ionic conductivity studies. 

**Figure 9:**
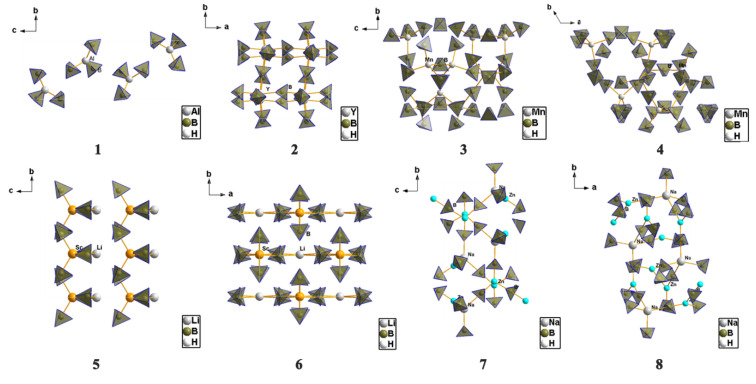
Selected examples of trivalent-, TM- (transition metal) and mixed-cation borohydrides. **1**—Al(BH_4_)_3_ (a-axis view); **2**—Y(BH_4_)_3_ (c-axis view); **3**—Mn(BH_4_)_2_ (a-axis view); **4**—Mn(BH_4_)_2_ (c-axis view); **5**—LiSc(BH_4_)_4_ (a-axis view); **6**—LiSc(BH_4_)_4_ (c-axis view); **7**—NaZn_2_(BH_4_)_5_ (a-axis view); **8**—NaZn_2_(BH_4_)_5_ (c-axis view). Color code: Sc—orange; Zn—blue.

Al(BH_4_)_3_ (**1** in Figure 9) is a liquid under normal conditions, but low temperature single-crystal XRD data shows two possible polymorphs with a transition temperature at ~190 K [83]. Single-crystal diffraction data by Aldridge et al. from 1997 shows that Al(BH_4_)_3_ crystallizes at 195 K in the orthorhombic *Pna2_1_* space group with the following unit cell parameters: *a* = 18.021(3) Å, *b* = 6.138(2) Å, and *c* = 6.199(1) Å. It consists of discrete Al(BH_4_)_3_ units, with a trigonal-planar geometry around the Al^3+^ center which coordinates the [BH_4_] tetrahedra via the tetrahedral edges. The Al-B bond lengths are in the range of 2.10(2)–2.14(2) Å, while the longer B-H distances for the B-H…Al bridges of 1.12(3)–1.14(4) Å are consistent with Al-H coordination [83].

*α*-Y(BH_4_)_3_, one of the most promising hydrogen storage candidates among transition metal borohydrides (**2** in Figure 9) was synthesized by cryo-milling of LiBH_4_ and YCl_3_. It crystallizes in the cubic *Pa*$\bar{3}$ space group with a lattice constant *a* = 10.8522(7)Å [65,68,69,70,71,72,73]. The structure has a Y^3+^ center in a highly distorted octahedral geometry being surrounded by six [BD_4_]^−^ tetrahedra (B-Y-B angles in the range 78.6(2)–160.9(1)°). The *α*-Y(BH_4_)_3_ converts at 10 MPa D_2_ and 475 K to the *fcc β-*Y(BH_4_)_3_ polymorph in the *Fm*$\bar{3}$*c* space group. 

*α*-Mn(BH_4_)_2_ (**3** in Figure 9) crystallizes in the *P3_1_12* space group and is isostructural with ζ-Mg(BH_4_)_2_, having the unit cell parameters *a* = 10.435(1)Å and *c* = 10.835(2)Å [189,190,191,192,193,194,195]. This represents the first crystal structure of a *3d*-metal tetrahydroborate. The *α*-Mn(BH_4_)_2_ polymorph has a stability range between 90 to 450 K. The structure bears structural similarities to Mg(BH_4_)_2_; the two independent Mn^2+^ centers are each tetrahedrally surrounded by four [BH_4_] units with Mn-B distances of 2.39–2.52 Å.

LiSc(BH_4_)_4_ can best be described as a Li^+^ salt of complex anion [Sc(BH_4_)_4_]^−^ that was evidenced by vibrational spectroscopy studies. It crystallizes in the tetragonal *P*$\bar{4}$*2c* space group, with a CuAlCl_4_ structural prototype and the following unit cell parameters: *a* = 6.076 Å and *c* = 12.034 Å (**5** in Figure 9). More specifically, it was obtained by Hageman et al. in 2008 by ball-milling processing of a 4:1 molar ratio LiBH_4_ and ScCl_3_ [181]. The central cation Sc^3+^ of the complex anion [Sc(BH_4_)_4_]^−^ is surrounded by four BH_4_ tetrahedra in a distorted tetrahedral coordination, with a Sc-B bond distance of 2.28(1)Å, which compares favorably to the DFT value predictions of 2.33 Å. The B-Sc-B angle has a spread distribution between 96.5(5)° and 124.4(5)°, suggestive of the deformed tetrahedral coordination. Each of the four [BH_4_] tetrahedra has three H atoms oriented towards the Sc center, so that the global coordination is 8 + 4: 8 Sc-H distances in the range 2.11–2.15 A and 4 Sc-H distances of 2.31 A, while the remaining B-H bond pointing outwards is responsible for the high B-H stretching frequencies of 2485–2498 cm^−1^. Moreover, the crystal structure contains disordered Li^+^ cations along the *z*-axis of the tetragonal cell, which in turn could make this mixed borohydride a suitable candidate for ionic conductivity applications [181]. 

NaZn_2_(BH_4_)_5_ (**7** in Figure 9) crystallizes in the monoclinic *P2_1_/c* space group, similar to the Li^+^ counterpart LiZn_2_(BH_4_)_5_, and it has no analogues to known inorganic structures. The unit cell parameters are: *a* = 9.397(2) A, *b* = 16.635(3)A, *c* = 9.136(2)A, and *β* = 112.66(2)° [41]. The structure consists of two independent Zn^2+^ cations that have almost a trigonal-planar coordination to three [BH_4_] tetrahedra (coordination number of Zn is 3), while the Na^+^ cation has a new, saddle-like coordination (coordination number of Na is 4) [41]. Interestingly, NaZn_2_(BH_4_)_5_ consists of two doubly penetrating 3D frameworks, with no covalent bonds between them—a structural motif found in coordination polymers. All [BH_4_] units are nearly linearly coordinated by two metal atoms, and the coordination occurs via the two opposite tetrahedral edges bridging two Zn^2+^ cations, or a Zn^2+^ and a Na^+^ cation. 

The hexagonal structure observed for many metal borohydrides can be attributed in part to an entropic term brought about by the vibrational amplitudes in BH_4_ units and the shortening of B-H bonds from 1.22 to under 1.1 Å [196,197]. Mixed-cation borohydrides give rise to a fascinating family of crystal structures, and have many times exhibited promising results regarding hydrogen release and reversible hydrogen uptake.

### 4.2. Stability of MBH

The most ionic and stable borohydrides have been studied since the 1940s, namely NaBH_4_, KBH_4_, RbBH_4_, and CsBH_4_; they are often stable in basic aqueous solution [165,166,167,168,169,198]. *Closo*-boranes are stable under neutral and acidic solutions. Being typically air- and moisture-sensitive compounds, their synthesis should be performed using Schlenk techniques or in the glove box, under inert (N_2_, Ar) atmosphere. Metal borohydrides are prone to adsorbing water, with which they react upon heating to release hydrogen. Some borohydrides of low electronegative elements are stable at RT, and even more so in the form of their hydrates. High electronegative metals forming metal borohydrides are highly reactive even below RT, and produce a highly exothermic reaction with water (some even explode upon contact with moisture). The layered structure of LiBH_4_ is non-trivial and shows local distortion of the polymorphs, which defines their stability and plays a role in corresponding phase transitions [199]. Theoretical computations based on first-principles study shed new light on the stability of the family of possible borohydride intermediates from LiBH_4_, namely Li_2_B_n_H_n_ (*n* = 5–12) [200]. 

Transition-metal borohydrides prepared by mechano-chemical methods are only stable when the ion itself is stable (d^0^, d^5^ and d^10^), that is, when it has no electrons in the d-orbital, at half-occupation, or when the d-shell is completely occupied [201]. However, lowering synthesis temperature to below −30 °C and using ammonia as stabilizing ligand, stable M(BH_4_)_2_(NH_3_)_6_ have been prepared in the case of M = Cr^2+^ (d^4^), Fe^2+^ (d^6^), and Co^2+^(d^7^). These borohydrides are only stable in solution at low temperature, and they have not been isolated in pure, solid form, with a d^1^ exception to the rule coming from a molecular borohydride of Ti(BH_4_)_3_, which has limited stability below 0°C [202]. Some borohydrides like Zr(BH_4_)_4_ (sublimes at 29 °C) form quality single-crystals by CVD when stored in a closed vessel, due to its molecular structure [203,204,205,206,207,208,209,210]. 

Bi- or tri-metallic borohydrides can usually be prepared by a mechano-chemical route; however, some diethyl ether Et_2_O solvates have been prepared and are stable in solvated form, such as NaMn(BH_4_)_3_·0.5 Et_2_O [211]. Dimethyl sulfide S(CH_3_)_2_ has recently been used as a weak-coordinating solvent which eases borohydride removal from the products, avoiding formation of ternary chlorides/bimetallic borohydrides. This strategy allowed for the synthesis and stabilization of some rare earth metal borohydrides M(BH_4_)_3_·S(CH_3_)_2_, where M is Y, La, Ce, or Gd [72,212,213,214,215]. Formation of ternary chlorides leads to incomplete reaction in mechanochemical synthesis; for instance, if Zn(BH_4_)_2_ is targeted, a mixture of NaBH_4_ and ZnCl_2_ used as reagents can often lead to the formation of Na_2_ZnCl_4_ by-product, which blocks Zn^2+^ cation availability for the metathesis reaction (Equation (40)).

(40) $$ZnCl_{2}+2\;NaCl⟹\;Na_{2}ZnCl_{4}$$

Other approaches utilize bulky-cation-stabilized precursors like [^n^Bu_4_N][Y(BH_4_)_4_] to produce less-stable borohydrides like LiZn_2_(BH_4_)_5_ [216,217].

### 4.3. Multi-Cationic Borohydrides

The first reported mixed alkali metal borohydride, LiK(BH_4_)_2_, was observed to form when a mixture of LiBH_4_ and KBH_4_ was mixed and heated to 125 °C [218]. This intermediate compound was fully characterized, including by crystal structure determination: Li^+^ coordinates to four [BH_4_] units, while K^+^ coordinates seven [BH_4_] tetrahedral unit. The decomposition temperature of LiK(BH_4_)_2_ is almost half of that of individual parent alkali-metal borohydrides.

Other mixed cation borohydrides are metastable, such as NaK(BH_4_)_2_; upon cooling to RT, the mixed metal borohydride separates into the monomeric borohydrides within one day [219]. The certainty of its formation by heating the system NaBH_4_–KBH_4_ was confirmed by an increase in the lattice parameter *a*, and the higher potassium content [220]. However, it was later proved that the mixed borohydride NaK(BH_4_)_2_ is not stoichiometric, but a solid solution of approximate formula Na_p_K_1-p_BH_4_ (0 < *p* < 1), and only stable at elevated temperatures (200–450 °C) [221].

Recently, a series of bimetallic borohydrides of alkali metal with alkaline earth, *d*- or *f*-block metals has been reported, containing the more electropositive alkali metal with a countercharge brought about by the complex anion [21,22,23,24,25,26,27,222,223]. K_2_Mg(BH_4_)_4_ and K_3_Mg(BH_4_)_5_ have isolated [Mg(BH_4_)_4_]^2−^ tetrahedra serving as complex anions, while the metal alkali K^+^ ensures the charge neutrality of the framework [224]. M^I^M^II^(BH_4_)_3_ (M^I^ = K, Rb, Cs; M^II^ = Ca, Sr, Sm) of perovskite-type 3D-networks have the divalent metal in octahedral coordination via κ^2^-bridging BH_4_ units, with the alkali metal cuboctahedrally surrounded by twelve BH_4_ units [185,225,226,227]. This perovskite-type structure is best stabilized by alkali-earth metals of smaller radii (Ca^2+^, 1.14 Å) than heavier analogues (Sr^2+^, 1.32 Å), a trend which is more apparent during heating [21,22,23,24,25,26,27,225,226].

Given the size-match between ammonium cation NH_4_^+^ (1,48 Å) and alkaline metals ionic radii K^+^ (1.38 Å) and Rb (1.52 Å), a family of bi-cationic borohydrides (NH_4_)_x_M(BH_4_)_y_ was produced when NH_4_BH_4_ was mixed with M(BH_4_)_p_ (M = Li, Na, Mg, Al, Y, Mn, Gd). These novel borohydrides exhibit high energy densities of up to 24.5 wt.% hydrogen [222].

### 4.4. Anion Substitution of MBH: From Light to Heavy Halides and Pseudo-Halide Substitution

Substitution of BH_4_^−^ was reported for LiBH_4_-LiCl [228], with this mixture actually forming a partial solid solution of anticipated formula Li(BH_4_)_1-p_Cl_p_ (0 < *p* < 1), an aspect which hindered further purification of the borohydride. The superionic HT-LiBH_4_ is formed at ~110 °C (108 °C), but this phase transition could occur at even lower temperatures possibly due to the formation of Li(BH_4_)_1-p_Cl_p_ during ball-milling [89,228]. Easier substitution has been confirmed for halide anions (F^−^, Cl^−^, Br^−^, I^−^) and it allowed for fine-tuning of the unit cell parameters and eventually to physical properties related to the crystallographic system and strain [229]. The shrinkage or enlargement of the unit cell volume mirrors the size of the anions: F^−^ (1.33 Å) < Cl^−^ (1.81 Å) < Br^−^ (1.96 Å) < BH_4_^−^ (2.05 Å) < I^−^ (2.20 Å) [153,230]. 

Depending on the crystallographic system and local environment, some polymorphs undergo anion substitution more easily than others, as shown by HT-LiBH_4_ and β-Mg(BH_4_)_2_ [231,232]. If both components of the system MBH_4_-MX (M-alkali metal, X- halide) are isostructural, anion substitution may occur both ways, such that two solid solutions can form: LiBH_4_–LiBr, LiBH_4_-LiI, or NaBH_4_-NaCl. While heavier halides (Br^−^, I^−^) stabilize the hydride, hindering hydrogen release at lower temperature, they lower the energy of hydrogen uptake during rehydrogenation [233,234,235,236]. While o-LiBH_4_ (orthorhombic) shows no conduction at RT, its HT-polymorph, HT-LiBH_4_ (hexagonal, stable at t > 110 °C), exhibits fast lithium-ion conductivity that is enhanced upon iodide anion I^−^ substitution of 25% with respect to starting BH_4_^−^ units, in Li[BH_4_]_0.75_I_0.25_ (Equation (41)) [237,238]. J. Rude et al. showed through usage of synchrotron radiation powder X-ray diffraction (SR-PXD) and attenuated total reflectance infrared spectroscopy (ATR-IR) that two solid solutions of hexagonal structure (just like HT-LiBH_4_ and β-LiI) form in the system LiBH_4_-LiI, which combine into one upon heating. The solid solutions of composition Li(BH_4_)_1-p_I_p_ (0 < *p* < 0.62) have been found to be stable over a 300-degree temperature range, and after four hydrogen release/uptake cycles they retained 68% of expected hydrogen capacity, in contrast to only 25% retained by pristine LiBH_4_.

(41) $$LT-LiBH_{4}\;\overset{\Delta }{\Rightarrow }\;HT-LiBH_{4}\overset{LiI}{\Rightarrow }Li{\left(BH_{4}\right)}_{0.75}I_{0.25}$$

Anion substitution has also been expanded to include amide -NH_2_- anions, and these boron–nitrogen species are promising from a hydrogen storage viewpoint and for properties of fast ion conductivities, as seen above in the case of halide substitution.

### 4.5. Stabilization of M(BH_4_)_x_ by Coordination of Neutral Molecules (NH_3_, N_2_H_4_, H_2_O, (CH_3_)_2_S)

While exhibiting a rich and fascinating structural diversity akin to that of metal organic frameworks (MOF), the metal borohydrides do often run into stability problems: they can be obtained solely by mechano-chemical synthesis, are stable only at low temperature, are only stable in solution or as solid solutions. These shortcomings can be overcome by neutral molecule coordination, enhancing their stability. Among these, N-containing ligands are particularly interesting because they can bind hydrogen, and could interact with the Lewis acidic boron through a frustrated Lewis pair (FLP)-type interaction: B-H^γ−^…^γ+^H-N. In fact, the shortest distance of 1.85 A has been reported for Y(BH_4_)_3_·7NH_3_, between the complex cation [Y(NH_3_)_7_]^3+^ and the borohydride anion BH_4_^−^. These interactions facilitate heterolytic E-H bond cleavage (E = B, N) to produce molecular H_2_, thus becoming attractive options for hydrogenation studies. Moreover, this inherent instability caused by H…H interactions gives rise to structural flexibility and variability. The solvent should behave as a Lewis base, containing highly electronegative atoms: N, O, S. In this regard, the most studied ligands are N-based ligands, such as ammonia NH_3_, hydrazine H_2_N-NH_2_, and ammonia–borane NH_3_BH_3_. S-based ligands (such as S(CH_3_)_2_, dimethyl sulfide) are more desirable than O-based ones (diethyl ether (C_2_H_5_)_2_O, tetrahydrofuran C_4_H_8_O, and water H_2_O), because M-S bonds are longer than M-O bonds and thus the S-ligand could potentially be easier to remove. 

A dihydrate adduct has been isolated in the case of NaBH_4_·2H_2_O crystallohydrate, where Na+ has octahedral coordination to four [BH_4_] units and two H_2_O molecules bridging two Na^+^ (space group: orthorhombic, *Pbca*) [153,239]. NaBH_4_·2H_2_O loses the two water molecules upon mild heating at 40 °C, and then slowly starts to release hydrogen (Equation (42)).

(42) $$NaBH_{4}\cdot 2H_{2}O\overset{40\;{}^{\circ}C}{\Rightarrow }\;NaBH_{4}+2\;H_{2}O\overset{40\;{}^{\circ}C}{\Rightarrow }NaBO_{2}+4\;H_{2}↑$$

Monohydrates of lithium and calcium borohydrides have also been reported: LiBH_4_·H_2_O and Ca(BH_4_)_2_·H_2_O [240].

A large class of ammine single-metal borohydrides M(BH_4_)_x_·*p*NH_3_ and ammine bimetallic borohydrides M^I^M^II^(BH_4_)_3_·*p*NH_3_ have been synthesized and structurally characterized, containing variable solvent molecules, in the range p = 1–8 (Equation (43)) [202,241,242].

(43) $$M{\left(BH_{4}\right)}_{x}+p\;NH_{3}⟹\;M{\left(BH_{4}\right)}_{x}\cdot pNH_{3}\;\;$$

For instance, LiBH_4_·NH_3_, the only alkali metal borohydride stable at RT as an ammine complex [243], Mg(BH_4_)_2_·*p*NH_3_ with *p* = 1,2,3, and 6 [244,245,246], as well as octa-ammine complex Zr(BH_4_)_2_·8NH_3_ [247], have been prepared and studied. For instance, the di-solvate Mg(BH_4_)_2_·2NH_3_ can be written in its molecular formula [Mg(BH_4_)_2_(NH_3_)_2_], with Mg^2+^ coordinating in a tetrahedral fashion two BH_4_^−^ units and two ammonia molecules [245]. Moreover, whereas only ions with electron configuration d^0^, d^5^, and d^10^ (with very few exceptions) could be obtained via the ball-milling technique, various other TM borohydride configurations (TM = transition metal) could be obtained as adducts due to enhanced stability brought about by NH_3_ coordination: d^1^ (Ti^2+^), d^2^ (V^3+^), d^6^ (Fe^2+^), or d^7^ (Co^2+^) [202].

Depending on the number of coordinating ligands, the thermal decomposition of ammine metal borohydrides can follow different pathways, as shown in Equation (44) [245].

(44) $$Mg{\left(BH_{4}\right)}_{2}\cdot 6NH_{3}\;\overset{\Delta ,-4\;NH_{3}}{\Rightarrow }\;\;Mg{\left(BH_{4}\right)}_{2}\cdot 2NH_{3}\;\overset{\Delta }{\Rightarrow }\;MgH_{2}+2\;BN+6\;H_{2}$$

The hexaamine complex Mg(BH_4_)_2_·6NH_3_ produces by thermal decomposition Mg(BH_4_)_2_·2NH_3_, which upon further heating releases irreversibly H_2_ and produces traces of BN as the sole crystalline phase. Rehydrogenation attempts at 250 °C for 60 h led to no amounts of Mg(BH_4_)_2_ [248]. 

A similar hexaammine complex Ca(BH_4_)_2_·6NH_3_ was produced by solid–gas reaction between Ca(BH_4_)_2_ and NH_3_(g) [249], and it is different from the solution synthesis used to prepare Mg(BH_4_)_2_·6NH_3_ (Equations (45)–(48)) [245].

(45) $$Ca{\left(BH_{4}\right)}_{2}{}_{\left(s\right)}+6\;NH_{3}{}_{\left(g\right)}⟹Ca{\left(BH_{4}\right)}_{2}\cdot 6NH_{3}\;\overset{\Delta ,20\;min,\;-2\;NH_{3},\;\;Ar}{\Rightarrow }\;\;Ca{\left(BH_{4}\right)}_{2}\cdot 4NH_{3}\;\;$$

(46) $$Ca{\left(BH_{4}\right)}_{2}\cdot 4NH_{3}\overset{87\;{}^{\circ}C,-2NH_{3},\;\;Ar}{\Rightarrow }Ca{\left(BH_{4}\right)}_{2}\cdot 2NH_{3}\overset{162\;{}^{\circ}C,-NH_{3},\;\;Ar}{\Rightarrow }Ca{\left(BH_{4}\right)}_{2}\cdot NH_{3\;}$$

(47) $$Ca{\left(BH_{4}\right)}_{2}\cdot NH_{3}\;\overset{230\;{}^{\circ}C,-NH_{3},\;\;Ar}{\Rightarrow }Ca{\left(BH_{4}\right)}_{2}$$

(48) $$Ca{\left(BH_{4}\right)}_{2}\cdot 2NH_{3}\overset{190-500\;{}^{\circ}C}{\Rightarrow }\;\frac{1}{4}\;\;Ca{\left(BH_{4}\right)}_{2}+\frac{1}{4}\;Ca_{3}{\left(BN_{2}\right)}_{2}+BN+6H_{2}$$

Reactions (45)–(47) were performed under Ar flow, when NH_3_ was released. Running the reaction in a closed vessel, no NH_3_ evolution was detected and instead a sudden dehydrogenation reaction with onset at 190 °C took place (Equation (48)). The final dehydrogenated species was assigned based on ^11^B NMR spectra, consistent with an overall hydrogen storage capacity of Ca(BH_4_)_2_·2NH_3_ of 12.3 wt.%. This diammoniate system was not reversible, as no rehydrogenation occurred during attempts at 50-bar H_2_ and 20–300 °C [249].

Coordination of H_2_N-NH_2_ (hydrazine) to metal borohydrides produces M(BH_4_)_x_·nN_2_H_4_ (M = Li, Na, Mg), out of which the LiBH_4_.N_2_H_4_ shows a remarkably high 13 wt.% H_2_ released at 140 °C when using Fe-B catalyst [250,251].

**Figure 10:**
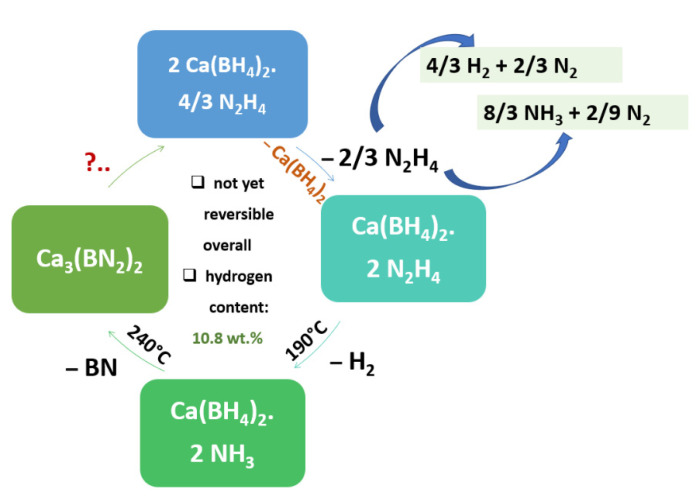
Overview of dehydrogenation pathways in Ca(BH_4_)_2_·4/3N_2_H_4_ (10.8 wt.% H_2_); the system is not yet reversible [252].

Ca(BH_4_)_2_ produces hydrazinates of composition Ca(BH_4_)_2_·nN_2_H_4_ (n = 1, 4/3, 2, 3) that store 8.8 wt.% H_2_ (n = 2), 9.2 wt.% H_2_ (n = 1) and 10.8 wt.% H_2_ (n = 4/3) (Figure 10). Z. Li et al. propose a dehydrogenation pathway involving H(δ+)…H(δ-) interaction [249] and NH_3_-mediated mechanisms [252]. The dehydrogenation temperature was reduced from 240 °C to 140 °C by utilizing an Fe-based catalyst (2–5 mol% FeCl_3_); however, the Fe^3+^ seemed to partially catalyze decomposition of hydrazine as well, as NH_3_ was found among the gaseous products (Equation (49)).

(49) $$CaCl_{2}+2\;NaBH_{4}\overset{BM,\;\;THF,-2NaCl}{\Rightarrow }Ca{\left(BH_{4}\right)}_{2}\overset{n\;N_{2}H_{4}}{\Rightarrow }Ca{\left(BH_{4}\right)}_{2}\cdot nN_{2}H_{4};n=1,\frac{4}{3},2,\;3\;\;\;$$

Hydrazine itself (H_2_N-NH_2_) has two competitive decomposition pathways, leading to either (2 H_2_ + N_2_), or (4/3 NH_3_ + 1/3 N_2_); at least one side-reaction thus leads to enhanced hydrogen storage capacity (Figure 10). However, this system has not achieved reversibility yet and research is due to regenerate Ca(BH_4_)_2_ from spent Ca_3_(BN_2_)_2_. 

Ammonium-substituted MBH were also produced via the cryo-mechanochemical route for a series of alkali metal (Li^+^, Na^+^), alkali-earth metals (Mg^2+^, Ca^2+^, Sr^2+^), and TM- and RE-borohydrides (Mn^2+^, Y^3+^, La^3+^, Gd^3+^) (Equation (50)) [222].

(50) $$x\;NH_{4}BH_{4}+y\;M{\left(BH_{4}\right)}_{m}\;\overset{-196\;{}^{\circ}C}{\Rightarrow }\;\;{\left(NH_{4}\right)}_{x}M_{y}{\left(BH_{4}\right)}_{x+my}$$

These ammonium borohydrides show decomposition in the temperature range 30–60 °C and usually release toxic diborane B_2_H_6_, along with dihydrogen H_2_ (Equation (51)).

(51) $${\left(NH_{4}\right)}_{3}Mg{\left(BH_{4}\right)}_{5}\overset{35\;{}^{\circ}C,-B_{2}H_{6}.-2H_{2}}{\Rightarrow }\;\;\left(NH_{4}\right)Mg{\left(BH_{4}\right)}_{3}\cdot 2NH_{3}\overset{40\;{}^{\circ}C.-H_{2}}{\Rightarrow }Mg{\left(BH_{4}\right)}_{2}\cdot 2NH_{3}\cdot NH_{3}BH_{3}$$

However, not all ammino-stabilized ammonium borohydrides release diborane upon heating; (NH_4_)Ca(BH_4_)_3_ decomposes to release an ammonia–borane adduct $Ca{\left(BH_{4}\right)}_{2}\cdot NH_{3}BH_{3}$ and hydrogen in the first step, producing *β*-Ca(BH_4_)_2_ upon further heating and loss of a molecule of ammonia borane NH_3_BH_3_. Interestingly, H_2_ is only released during the first, lower-temperature decomposition step; (NH_4_)Li(BH_4_)_2_ follows a similar decomposition pathway to release hydrogen (Equation (52)) [222].

(52) $$\left(NH_{4}\right)Ca{\left(BH_{4}\right)}_{3}\overset{111\;{}^{\circ}C,\;-H_{2}}{\Rightarrow }\;\;Ca{\left(BH_{4}\right)}_{2}\cdot NH_{3}BH_{3}\overset{162\;{}^{\circ}C.-NH_{3}BH_{3}}{\Rightarrow }\beta -Ca{\left(BH_{4}\right)}_{3}$$

## 5. Physical and Chemical Properties of M(BH_4_)_x_

Ionic and covalent borohydrides will intrinsically have different properties. For a rough estimation, Pauling’s empirical rule and Hannay–Smith formulae have been employed to estimate the ionicity percentage in known and reported borohydrides. As the electropositivity of the metal increases, the bonding character is more ionic, such that the most ionic borohydrides are those of alkali metal [253]. While the main properties of metal borohydrides have been polarized by their high and potentially reversible hydrogen content, other studies have expanded their field of interest, including optical, magnetic, and semiconductor materials.

### 5.1. Electrochemistry of Metal Hydrides and M(BH_4_)_x_: Electrodes, Electrolytes (Li^+^, Mg^2+^, Ca^2+^), Complex Metal Hydrides

Due to their strong reducing character, metal borohydrides have recently been investigated as electrolytes in liquid and solid form, showing conductivities σ higher than 10^−4^ S/cm [21,22,23,24,25,26,27,254,255]. Solid-state electrolyte research reached new heights after the discovery that *o*-LiBH_4_ (*Pnma*, orthorhombic, ionic conductivity σ_Li+_ = 10^−8^ S/cm) transforms at 108–110 °C into another high temperature polymorph *h*-LiBH_4_ (*P6_3_mc*, hexagonal, σ_Li+_ = 10^−3^ at 120 °C, five orders of magnitude higher than that of *o*-LiBH_4_) (Table 2) [256,257,258,259,260,261]. As shown previously, several strategies have been employed to obtain RT-stable borohydrides with comparable ion conductivity: halide or halide-alike substitution in the complex anion (Li_4_(BH_4_)_3_I, Li_2_(BH_4_)(NH_2_)), nanoconfinement in mesoporous silica of LiBH_4_ or Li_4_(BH_4_)_3_I, or formation of rare-earth (RE)-containing bimetallic borohydrides Li(RE)(BH_4_)_3_Cl (RE = La, Ce, etc.) [262,263]. Ionic conductivity has been expanded by utilizing neutral ligands or larger closo-borate salts which prove to ease the cation migration and represent the state-of-the-art in research on borohydride materials as electrolytes.

**Figure 11:**
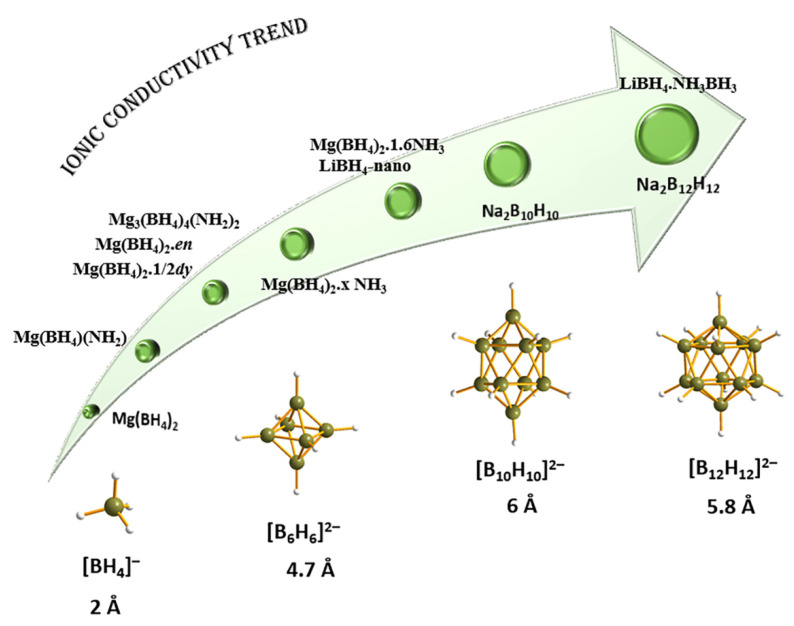
Boron–hydrogen speciation and influence on ionic conductivity of corresponding alkali and alkali-earth salts.

Most conductivity studies have been carried out regarding LiBH_4_ and Mg(BH_4_)_2_ complexes, especially on neutral ligand-stabilized solvates of these borohydrides. It becomes apparent that ionic radii increase must be an obstacle in front of ion migration, as Ca^2+^ in Ca(BH_4_)_2_ shows slow conductivity (r_Li+_ = 0.9 Å; r_Mg2+_ = 0.86 Å; r_Ca2+_ = 1.14 Å). It seems that ~26% increase in ionic radii from Li^+^ to Ca^2+^ would inhibit ion migration in the framework. The interaction charge framework is important and increases with the charge of the cation, but it is not essential for conductivity. Although they have the same charge (+2) of Mg^2+^ and Ca^2+^, respective borohydrides are different in terms of conductivity, with solvated Mg(BH_4_)_2_ showing real promise as an electrolyte candidate with an oxidation stability of 1.2 V (by CV, cyclic voltammetry) (Table 2) [272,275,276]. Recent trends in improving ionic conductivity have explored complexes with weaker coordinating ligands/counteranions, as is the case for CaB_12_H_12_ [277]. Despite their low electrochemical stability, borohydrides give rise to rich speciation: nido- (typical tetrahydroborate BH_4_^−^), planar- (B_6_H_6_^6−^), and closo (B_10_H_10_^2−^; B_12_H_12_^2−^), making their alkali (Li^+^, Na^+^, K^+^) and alkali-earth (Mg^2+^, Ca^2+^) salts particularly appealing in ion conduction applications [278,279,280,281]. The ionic conductivity trend correlates well with the size of the hydroborate species (Figure 11).

The closo-borates are currently under active research due to their rapid ion conduction, due in part to the open structure with channels for ionic conduction [281]. Both Li_2_B_12_H_12_ and Na_2_B_12_H_12_ exhibit high ionic conductivities of the order of magnitude of 10^−1^ S cm^−1^ which are beneficial for Na-battery electrolytes [257,258,259]. The superionicity found in these closo-salts could also originate from the high rotational mobility of the large B_12_H_12_^2−^ anion [259].

### 5.2. Optical and Magnetic Properties

As expected for RE (RE = rare earth) complexes, Eu(BH_4_)_2_·2THF has blue luminescence and an impressive quantum yield of ~75%, due to charge separation of Eu^2+^ centers by borohydride BH_4_^−^ anions, which avoid quenching. Similar favorable luminescent properties were found in Y(BH_4_)_2_·2THF [282]. The ionic bond Eu^2+^–BH_4_^−^ allows d → f emission in the blue region of the spectrum. A series of mixed metal borohydrides of perovskite-type structure, such as KYb(BH_4_)_3_ and CsEu(BH_4_)_3_, have also been investigated, with a red shift in the latter compared to parent Eu(BH_4_)_2_·2THF [225,226]. Avoidance of quenching has been proven by addition of a 5% Eu^2+^ in CsEu(BH_4_)_3_, which showed similar luminescence properties regardless of [Eu^2+^] doping concentration [225,226].

### 5.3. M(BH_4_)_x_ as Semi- and Superconductors

Borohydrides of perovskite structure could be fine-tuned for photovoltaics, as their typical band-gap (>5 eV) could be lowered to ~1.5 eV at RT in the particular case of CsPb(BH_4_)_3_, which has semiconductor behavior. The reasoning for this seems to lie in the partial covalent bonding in the Pb(BH_4_)_3_ framework, supported by the p(Pb) states present in the conduction band edge [225].

Superconductivity has been achieved for metal hydrides at very high pressures, exceeding 100 GPa, but providing a solid proof-of-concept. While the lanthanum-based borohydride LaBH_8_ is not yet confirmed experimentally, computations predict it to be a superconductor and stable at a considerably lower pressure of about 40 GPa [283,284,285]. However, recent reports (2021) suggest that under pressure (12 GPa) a ternary alkali borohydride KB_2_H_8_ shows phonon-mediated high-temperature superconductivity [226]. 

### 5.4. CO_2_ Capture

Reaction of MBH with CO_2_(g) can be used for carbon dioxide sequestration/removal, or to produce derivatives like formates (Equation (53)) [286,287].

(53) $$MBH{\left(HCOO\right)}_{3}\left(DME\right)\overset{3\;CO_{2},\;DME}{⇐}\;\;MBH_{4}\overset{\Delta ,\;\;\;2\;CO_{2}}{\Rightarrow }\;MBO\left(HCOO\right)(OCH_{3});\;\;M=Li,\;Na$$

The reaction with CO_2_ can follow different pathways, depending on temperature and solvent used (DME, dimethoxyethane), which adds to the growing interest in CO_2_ capture globally [288,289]. LiBH_4_ and NaBH_4_ reaction with CO_2_ was also monitored in non-catalytic, solvent-free conditions, when IRGA (infrared gas analysis), FTIR, ^11^B NMR, and ^13^C NMR confirmed the presence, respectively, of borate, formate, and methoxy species [290].

Reaction of KBH_4_ under solvent-free ball-milling with solid CO_2_ leads to a formylhydroborate species K[H_x_B(OCHO)_4-x_] (x = 1–3) [291]. The synthesis of this interesting substituted borate species follows a three-step, consecutive nucleophilic attachment of the borohydride H^−^ on the electron-deficient carbon of CO_2_ molecules (Equation (54)).

(54) $$\;\;KBH_{4}\;\overset{CO_{2}}{\Rightarrow }K\left[H_{3}B\left(OCHO\right)\right]\;\overset{CO_{2}}{\Rightarrow }K\left[H_{2}B{\left(OCHO\right)}_{2}\right]\overset{CO_{2}}{\Rightarrow }K\left[HB{\left(OCHO\right)}_{3}\right]$$

While reaction of MBH with H_2_O usually follows the pattern seen in Equations (42) and (55), variations including running the reactions under small CO_2_ amounts have proven fruitful in synthesizing novel substituted metal borohydrides [292].

(55) $$MBH_{4}+\left(m+2\right)\;H_{2}O⟹MBO_{2}\cdot m\;H_{2}O+4\;H_{2}$$

Contrary to the Li^+^ and Na^+^ counterparts, KBH_4_ borohydride produces under CO_2_-promoted hydrolysis a metal complex still retaining the [BH_4_^−^] moiety: K_9_[B_4_O_5_(OH)_4_]_3_(CO_3_)(BH_4_)·7H_2_O. This complex was investigated by XRD, DSC, TGA, Raman, and IR spectroscopies and fully characterized by solving its single crystal structure, showing a hexagonal crystal structure belonging to the P-62c space group, and high hydrogen gravimetric content. The reaction proposed by Filinchuk et al. is complex and leads to potassium carbonate as by-product (Equation (56)), along with a large amount of hydrogen, so that it can be regarded as having a double utility: removal of CO_2_ from car exhaust gases, and efficient conversion of CO_2_ to a clean fuel, H_2_ [286]:

(56) $$\;13KBH_{4}+37H_{2}O+3CO_{2}\;⟹K_{9}{\left[B_{4}O_{5}{\left(OH\right)}_{4}\right]}_{3}\left(CO_{3}\right)\left(BH_{4}\right)\cdot 7H_{2}O+2K_{2}CO_{3}+48H_{2}$$

The porous polymorph of *γ*-Mg(BH_4_)_2_ adsorbs CO_2_ under very mid, near-RT conditions and also yields formate and methoxy products, proving suitable for CO_2_ sequestration. The highest CO_2_ uptake of 12 mol/kg was recorded after 7 days of exposure at 1-bar CO_2_ and 30 °C, and was attributed to the very porous nature of magnesium borohydride polymorph, which features a very large specific surface area [293].

## 6. M(BH_4_)_x_ as Hydrogen Storage Materials

### 6.1. Thermodynamic Properties of MBH

Thermal decomposition of metal borohydrides is directly related to the electronegativity of the M metal, which roughly displays a clear decrease in decomposition temperature with the increase in M electronegativity [294]. 

Pauling hypothesized that the energy E(A-B) of a heteroatomic bond (A ≠ B) relates to the difference in electron affinities of A and B by Equation (57), where the second term can be approximated in the case of compounds MR_n_ (R-anion) by *−*Δ*H_f_/n.*

(57) $$\chi_{A}-\chi_{B}\propto \sqrt{E_{A-B}-\frac{E_{A-A}+E_{B-B}}{2}}\;\;\propto \;\;\sqrt{\frac{-\Delta H_{f}}{n}}$$

The decomposition temperature (T_d_, K, and t_d_, °C; T_d_[K] = t_d_(°C) + 273.15) can be correlated to the Pauling electronegativity of the cation [295,296,297,298,299]. The empirical formula (Equation (58)) used to determine T_d_ was found to be [300]:

(58) $$T_{d}\left[K\right]=175.914{\left(\chi_{P}-3.11\right)}^{2}\;\;or\;t_{d}\left({}^{\circ}C\right)=175.914\;\chi_{P}{}^{2}-1094.2\;\chi_{P}+1428.3\;$$

**Figure 12:**
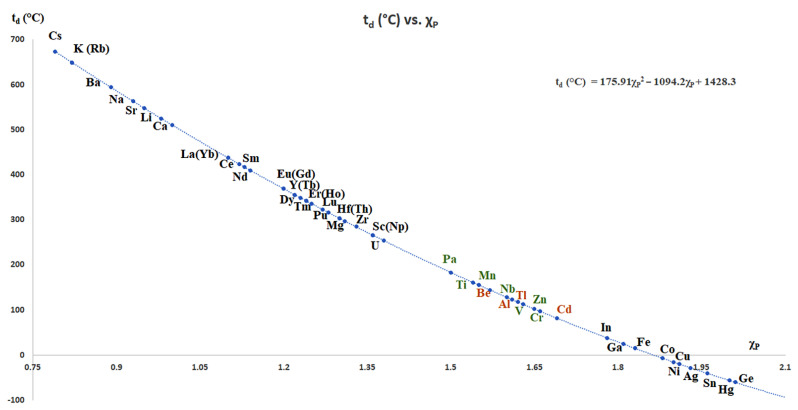
Decomposition temperature of M(BH_4_)_x_ dependent on cation (*Pauling) electronegativity following a van’t Hoff equation plot. In green are highlighted elements forming borohydrides with predicted decomposition temperature 50–150 °C, suitable for mobile application. Very toxic elements are in red [295,296,297,298,299].

The van’t Hoff equation adapted for decomposition of metal borohydrides (Equation (58)) contains the factor 3.11, which can be regarded as the electron attractive coefficient of borohydride anion [BH_4_]^−^, while 175.914 is a coefficient containing entropy values of decomposition (oversimplified as being constant for all compounds, in a first approximation) (Figure 12).

First-principles calculations led to the estimation of formation enthalpies for metal borohydrides, with an absolute error of only 10.4 kJ/mol (Equation (33)) [295,296,297,298,299].

(59) $$\Delta H_{boro}=248.7\;\chi_{P}-390.8\;\;\;\;\;\;\;;\;T_{d}=175.914{\left(\chi_{P}-3.11\right)}^{2}$$

**Figure 13:**
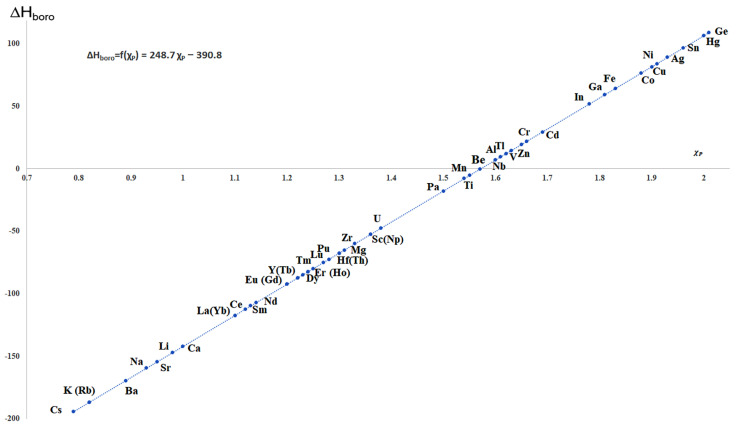
Formation enthalpies ΔH^0^_f_ (ΔH_boro_, kJ/mol BH_4_) for metal borohydrides that are either reported or believed to exist (Figure 2).

A representative plot for metal borohydrides presented in Figure 2 is given in Figure 13, where a linear correlation between enthalpy and electronegativity $\chi_{P}$ is confirmed. 

Pauling electronegativities are preferred because when inferring Mulliken scaling ($\chi_{M}$), the values are fitted for ΔH_boro_ with a higher error (13.1 kJ/mol) and moreover the enthalpies for Cu^+^ and Zn^2+^ borohydrides are almost the same (Mulliken electronegativities: 4.48 eV and 4.45 eV, respectively), which cannot explain the differences observed experimentally between the two [294,301,302]. Based on Equation (59), it can be inferred that for metal borohydrides having ΔH^0^_f_ ≥ 0, that is for $\chi_{P}\geq 1.57$, the borohydride would have an endothermic formation enthalpy and could be considered unstable (Table 3). For selected M(BH_4_)_x_ the free Gibbs energy ΔG has also been computed. Group I and II metal borohydrides have been the most investigated regarding thermodynamic data, and the following suggestive formulae were found (in kJ/mol formula unit); $\Delta G_{LiBH_{4}}=-222466.273+751.660356\;T-124.7\;TlnT$ [296], $\Delta G_{NaBH_{4}}=-217735+693\;T-119.233\;TlnT$ [296], $\Delta G_{NaBH_{4}}=16926-21.756\;T$ [297], $\Delta G_{KBH_{4}}=19176-21.841\;T$ [296], $\Delta G_{\alpha -Mg{\left(BH_{4}\right)}_{2}}=-222624.9+158.46145\;T-35.22138\;TlnT-0.035975\;T^{2}$ [298], $\Delta G_{\beta -Mg{\left(BH_{4}\right)}_{2}}=12954.437-26.4266\;T$ [298]. 

Screening the known borohydrides, one might expect the following elements to form unstable borohydrides: Cu, Ag, (Be), Cr, Fe, Ni, Co, Zn, Cd, Hg, Al, V, Ga, Nb, In, Ge, and Sn. Many of these are known, but either they are stable at very low temperatures or they need other techniques for stabilizations, i.e., formation of adducts with neutral molecules (Table 2). 

Many borohydrides follow a decomposition scheme leading to production of B_2_H_6_ and H_2_ in a 1:1 molar ratio (Equation (60)). These pathways that lead to production of diborane (toxic gas) will be, unfortunately, not useful for expanding the class of hydrogen storage material because they lead to a loss of the boron source [300].

(60) $$Cd{\left(BH_{4}\right)}_{2}\;\overset{75\;{}^{\circ}C}{\Rightarrow }\;Cd+B_{2}H_{6}+H_{2}$$

### 6.2. Destabilizing Methods for Complex Metal Borohydrides

*Nanoconfinement* is a valuable method for the synthesis of nano-sized MBH. This route can be utilized to lower energy barriers and speed up kinetics. Nanoscaffolds can be engineered to have highly advantageous textural properties: tunable pore size (D_p_), as well as high pore volume (V_p_) and specific surface area (SSA), which in turn will greatly enhance the kinetics of hydrogen release/uptake. These *recyclability enhancements* will be described for representative metal borohydride systems and their composites. Anion substitution in complex metal hydrides can lead to system destabilization: TiF_3_ and TiCl_3_ were investigated in NaBH_4_ and showed a 6.6 kJ/mol decrease in decomposition enthalpy, by altering hydrogen release and uptake [303]. The replacement of H^−^ by F^−^ was investigated in LiBH_4_ as well, showing significant theoretical thermodynamic improvements [304]. Nanoconfinement improvements brought about by size-restriction are discussed in relation to their bulk state in the following chapter (Section 7). Some volatile borohydrides such as Zr(BH_4_)_4_ could be embedded into mesoporous silica (MCM-48 type) to show enhanced stability towards air and moisture, and more importantly, to retain Zr(BH_4_)_4_ inside siloxanic pores, thus mitigating the high volatility issue of neat Zr(IV) borohydride.

*Thermal destabilization* by utilizing mixtures of metal borohydrides has proven to be an effective technique [305]. Coupled dehydrogenation of a physical mixture (1 − x)LiBH_4_ + xCa(BH_4_)_2_ comprised of *o-*LiBH_4_ and *α-/γ-*Ca(BH_4_)_2_ (synchrotron XRD patterns) also led to enhanced ionic conductivity of the resulting *cubic-*CaH_2_. Moreover, it seems that the ionic conductivity may be solely due to Li^+^ and irrespective of Ca^2+^ content, as shown by samples (1 − x)LiBH_4_+xCa(BH_4_)_2_ with similar ionic conductivity to that of pure ball-milled LiBH_4_ [305]. Further investigations by first-principles computations of a similarly coupled system by Ozolins et al. have shown that the decomposition reaction yields the complex intermediate Li_2_B_12_H_12_ alongside the anticipated CaH_2_ and hydrogen H_2_, with an endothermic enthalpy of 37.9 kJ/mol and $6.7\;\mathrm{wt}.\%\;H_{2}$ (Equation (61)) [306].

(61) $$\;5Ca{\left(BH_{4}\right)}_{2}+2LiBH_{4}\;\overset{83\;{}^{\circ}C}{\Rightarrow }Li_{2}B_{12}H_{12}+5CaH_{2}+13H_{2};\;\;\;\;\;\;\;\;\;\;\;\;\;\;\;\;\Delta H^{300K}=37.9\frac{\mathrm{kJ}}{\mathrm{mol}}$$

Using Mg(BH_4_)_2_ with either LiBH_4_ (Equation (62); 8.4 wt.%H_2_) or Ca(BH_4_)_2_ (Equation (63); 7.7 wt.%H_2_) produces similar chemical reactions, but at negative temperatures which therefore would hinder their usage under ambient conditions.

(62) $$\;5Mg{\left(BH_{4}\right)}_{2}+2LiBH_{4}\;\overset{-29\;{}^{\circ}C}{\Rightarrow }Li_{2}B_{12}H_{12}+5MgH_{2}+13H_{2};\;\;\;\;\;\;\;\;\;\;\;\;\;\Delta H^{300K}=24.4\frac{\mathrm{kJ}}{\mathrm{mol}}$$

(63) $$\;5Mg{\left(BH_{4}\right)}_{2}+Ca{\left(BH_{4}\right)}_{2}\;\overset{-18\;{}^{\circ}C}{\Rightarrow }CaB_{12}H_{12}+5MgH_{2}+13H_{2};\;\;\;\;\;\;\;\;\;\Delta H^{300K}=25.7\frac{\mathrm{kJ}}{\mathrm{mol}}$$

Decomposition of group II metal dodecaborohydrides MB_12_H_12_ seems to occur around 300 °C (309 °C for MgB_12_H_12_ and 306 °C for CaB_12_H_12_), but the group I counterpart shows unfavorable thermodynamics (LiB_12_H_12_, ΔH = 116.7 kJ/mol, t_d_ = 696 °C) (Equations (64) and (65)).

(64) $$\;Li_{2}B_{12}H_{12}\;\overset{696\;{}^{\circ}C}{\Rightarrow }2LiH+12B+5H_{2};\;\;\;\;\;\;\;\;\;\;\;\;\;\;\;\;\;\;\;\;\;\;\;\;\;\;\;\;\;\;\;\;\;\;\;\Delta H^{300K}=116.7\frac{\mathrm{kJ}}{\mathrm{mol}}\;$$

(65) $$\;CaB_{12}H_{12}\;\overset{306\;{}^{\circ}C}{\Rightarrow }CaB_{6}+6B+6H_{2};\;\;\;\;\;\;\;\;\;\;\;\;\;\;\;\;\;\;\;\;\;\;\;\;\;\;\;\;\;\;\;\;\;\;\;\;\;\;\;\Delta H^{300K}=73.7\frac{\mathrm{kJ}}{\mathrm{mol}}$$

Further hydrogen release by reaction of [B_12_H_12_]^2−^ complex anion with the metal hydrides MH_2_ should lead to additional hydrogen release (Equations (66) and (67)). It is thermodynamically more favored the addition of CaH_2_ rather than MgH_2_ to CaB_12_H_12_, as the switch from MgH_2_ to CaH_2_ used for destabilization could lower the operating temperature of the system even more, from 144 °C to 86 °C [306].

(66) $$\;CaB_{12}H_{12}+3MgH_{2}\;\overset{144\;{}^{\circ}C}{\Rightarrow }3MgB_{2}+CaB_{6}+9H_{2};\;\;\;\;\;\;\;\;\;\;\;\;\;\;\;\Delta H^{300K}=53.2\frac{\mathrm{kJ}}{\mathrm{mol}}$$

(67) $$\;CaB_{12}H_{12}+CaH_{2}\;\overset{86\;{}^{\circ}C}{\Rightarrow }2CaB_{6}+7H_{2};\;\;\;\;\;\;\;\;\;\;\;\;\;\;\;\;\;\;\;\;\;\;\;\;\;\;\;\;\;\;\;\;\;\;\;\;\Delta H^{300K}=47.0\frac{\mathrm{kJ}}{\mathrm{mol}}$$

**Figure 14:**
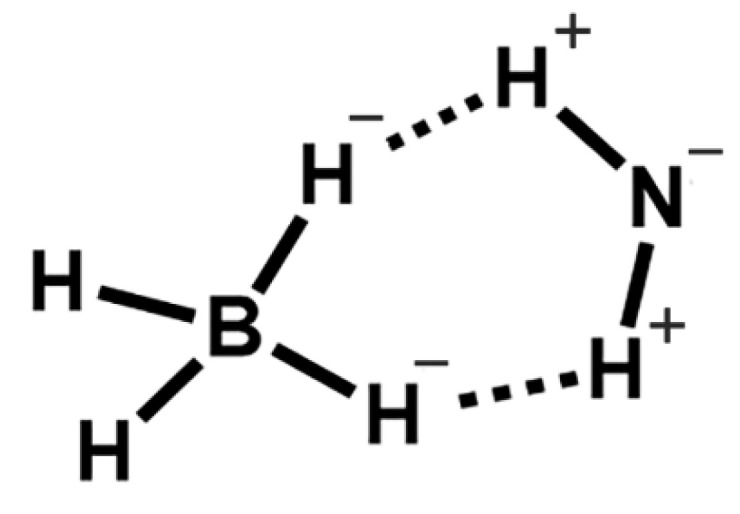
Dihydrogen bonding represented between borohydride [BH_4_]^−^ and amidic [NH_2_]^−^ moieties.

*Ca(BH_4_)_2_-M(NH_2_)_2_ destabilized system (M = Mg, Ca)* is a typical and representative system producing significant thermodynamic alteration for decomposition of Ca(BH_4_)_2_. Destabilization of Ca(BH_4_)_2_ was attempted with various group II amides (Mg(NH_2_)_2_, Ca(NH_2_)_2_) leading to mixed-metal-/metal nitridoborates $\left[Ca_{3}Mg_{6}{\left(BN_{2}\right)}_{6}\right]$ and $Ca_{9}{\left(BN_{2}\right)}_{6}$, respectively. The reasoning for such an outcome lies in the interaction of [BH_4_]^−^ and [NH_2_]^−^ anions, and more precisely in the B-H…H-N interaction of dihydrogen bonding type (Figure 14) [307,308]. 

While the dehydrogenation onset of bulk Ca(BH_4_)_2_ is roughly 320° (290_onset_-361_peak_), the binary system Ca(BH_4_)_2_-2Mg(NH_2_)_2_ (8.3 wt.% H_2_) and Ca(BH_4_)_2_-2Ca(NH_2_)_2_ (6.8 wt.% H_2_) starts to release hydrogen at 220 °C, so that a significant ΔT of 100 degrees is produced. Although some NH_3_ release was recorded, XRD study of the final dehydrogenated binary systems indicates Equations (68) and (69) as taking place when heating the samples below 480 °C [309].

(68) $$Ca{\left(BH_{4}\right)}_{2}+2Mg{\left(NH_{2}\right)}_{2}\;\;\overset{220-480\;{}^{\circ}C}{\Rightarrow }\frac{1}{3}\left[Ca_{3}Mg_{6}{\left(BN_{2}\right)}_{6}\right]+8H_{2}$$

(69) $$Ca{\left(BH_{4}\right)}_{2}+2Ca{\left(NH_{2}\right)}_{2}\;\;\overset{220-480\;{}^{\circ}C}{\Rightarrow }\frac{1}{3}\;Ca_{9}{\left(BN_{2}\right)}_{6}+8H_{2}$$

The binary systems were prepared by BM (ball-milling technique, 200 rpm, 5 h, Ar) while the nature of the evolved gases was assessed by MS-coupled TPD (temperature-programmed desorption) [309]. This pattern of destabilization has been reported as well for LiBH_4_-LiNH_2_ [310], NaBH_4_-NaNH_2_ [311], Ca(BH_4_)_2_-LiNH_2_ [312,313], and Ca(BH_4_)_2_-NaNH_2_ [313] systems, and they all led to new complex hydrides. 

Table 4 summarizes the main destabilization systems known for metal borohydrides; these include destabilization by metal, metal oxide, metal chloride, amides, and even metal borohydrides. The decomposition pathway is often altered by the molar ratio M(BH_4_)_x_: *destabilizing agent*. For instance, a clear dehydrogenation proposal (Equation (70)) was advanced by Aoki et al. for LiBH_4_-2LiNH_2_ [314], while others reported the dehydrogenation for LiBH_4_-LiNH_2_ without an explicit mechanism.

(70) $$\;LiBH_{4}+2LiNH_{2}\;\overset{249\;{}^{\circ}C}{\Rightarrow }\;Li_{3}BN_{2}+4H_{2}\;;\;\;\;\;\;\;\;\;\Delta H=23\frac{\mathrm{kJ}}{\mathrm{mol}H_{2}};\;\;\;\;11.9\;\mathrm{wt}.\%\;H_{2}$$

Somer et al. propose two possible reactions in the NaBH_4_:NaNH_2_ 1:1 and 1:2 systems (Equations (71) and (72)), with very different outcomes. The 1:1 system leads to the production of amorphous boron B and Na metal, whereas the 1:2 mixture leads to hydrogen evolution and a unique solid product, trisodium dinitridoborate Na_2_BN_2_ [311].

(71) $$NaBH_{4}+NaNH_{2}\;\overset{400\;{}^{\circ}C}{\Rightarrow }\;NaH+B+Na+\frac{1}{2}N_{2}+\frac{3}{2}H_{2}$$

(72) $$NaBH_{4}+2NaNH_{2}\;\overset{297\;{}^{\circ}C}{\Rightarrow }\;Na_{3}BN_{2}+4H_{2}$$

When LiH was mixed with γ-Mg(BH_4_)_2_ in various molar ratios (1:0.5, 1:0.97, and 1:1.89), it was found that the decomposition follows the one of pure products resulting from a metathetical reaction (Equation (73)). The sample 1 γ-Mg(BH_4_)_2_: 0.5 LiH showed the highest hydrogen storage capacity, essentially equal to the theoretical value [315].

(73) $$Mg{\left(BH_{4}\right)}_{2}+2LiH\;\overset{380-420\;{}^{\circ}C}{\Rightarrow }\;2LiBH_{4}+MgH_{2}$$

Destabilization of Ca(BH_4_)_2_ with LiNH_2_ in a 1:4 molar ratio yields a cubic phase of an anion-substituted borohydride [Ca(BH_4_)(NH_2_)], alongside a hydro-nitridoborate phase of Li_4_BN_3_H_10_. Upon heating to either 288 °C (5 h, undoped sample) or 178 °C (5 h, 5 wt.% CoCl_2_), the system 1 Ca(BH_4_)_2_: 4 LiNH_2_ releases 8 wt.% and 7 wt.% hydrogen, respectively (Equation (74)).

(74) $$Ca{\left(BH_{4}\right)}_{2}+4LiNH_{2}\overset{BM}{\Rightarrow }Li_{4}BN_{3}H_{10}+\left[Ca\left(BH_{4}\right)\left(NH_{2}\right)\right]\overset{288\;{}^{\circ}C,\;5\;h}{\Rightarrow }\frac{1}{4}LiCa_{4}{\left(BN_{2}\right)}_{3}+\frac{5}{4}Li_{3}BN_{2}+8H_{2}$$

DFT computation by Ozolins et al. showed various dehydrogenation pathways for binary borohydride–borohydride, borohydride–amide and borohydride–metal systems (Equation (75)) [306]. The system 5Mg(BH_4_)_2_ + 2LiBH_4_ (8 wt.% H_2_) has a decomposition temperature of −29 °C and is therefore ruled out from usage in mobility applications; however, the Ca^2+^-counterpart 5Ca(BH_4_)_2_ + 2LiBH_4_ (6.7 wt.% H_2_) has a reasonably low hydrogen release temperature of 83 °C [306].

(75) $$5M{\left(BH_{4}\right)}_{2}+2LiBH_{4}\Rightarrow Li_{2}B_{12}H_{12}+5MH_{2}+13H_{2}\;;\;\;\;\;\;M=Mg,\;Ca\;\;\;\;\;\;\;$$

Similarly, the system 5Mg(BH_4_)_2_ + Ca(BH_4_)_2_ with 7.7 wt.% H_2_ (theoretical) was predicted by first-principles computations to release H_2_ at −18 °C [306], but was actually found experimentally to only dehydrogenate around 150 °C, and the reaction was not reversible (Equation (76)) [316,317].

(76) $$5Mg{\left(BH_{4}\right)}_{2}+Ca{\left(BH_{4}\right)}_{2}\Rightarrow CaB_{12}H_{12}+5\;MgH_{2}+13H_{2}$$

Assuming that in composites of type 2Mg(BH_4_)_2_ + Ca(BH_4_)_2_ a temperature high enough is reached (above 306–309 °C, the decomposition temperature for plausible intermediates CaB_12_H_12_ and MgB_12_H_12_), one may consider that final decomposition products are those expected from individual borohydrides, so that the overall decomposition can be formulated as in Equations (77) and (78), with a theoretical hydrogen storage capacity of 10.5–12.75 wt.% [316,317,318].

(77) $$2Mg{\left(BH_{4}\right)}_{2}+Ca{\left(BH_{4}\right)}_{2}\Rightarrow \;\frac{1}{3}CaB_{6}+\frac{2}{3}CaH_{2}+4B+2MgH_{2}+\frac{28}{3}H_{2}\;;10.5\mathrm{wt}.\%H_{2}\;\;\;\;\;\;$$

(78) $$2Mg{\left(BH_{4}\right)}_{2}+Ca{\left(BH_{4}\right)}_{2}\Rightarrow \;\frac{1}{3}CaB_{6}+\frac{2}{3}CaH_{2}+2MgB_{2}+\frac{34}{3}H_{2}\;;\;\;\;12.75\mathrm{wt}.\%H_{2}\;\;\;\;\;\;\;\;\;\;\;\;$$

**Table 4:** Representative examples of destabilized borohydride systems: binary *borohydride–amide* systems M(BH_4_)_x_-RNH_2_, *borohydride–borohydride* systems M(BH_4_)_x_-R(BH_4_)_y_, *borohydride–chloride* M^I^BH_4_-MgCl_2_, *borohydride–TM oxide*, *borohydride–metal*.(t_d_: decomposition temperature).

System	Catalyst	t_d_ (°C)	Obs.	Reference
LiBH_4_–LiNH_2_	- via metastable Li_2_[BH_4_][NH_2_] and Li_4_[BH_4_][NH_2_]_3_	95_melt_-160_onset_-315_peak_, 230_mean_	5.8 wt.%H_2_; H_2_ major; small traces of NH_3_ (TPD-MS data)	[310]
LiBH_4_-2LiNH_2_	- (LiH possible intermediate)	249	7.8 wt.%H_2_ *LiBH_4_*: 75 kJ/molH_2_; *LiBH_4_-2LiNH_2_*: 23 kJ/molH_2_	[314]
*x* NaBH_4_–NaNH_2_ (*x* = 1,2,3,4)	- via α-/β- Na_2_[BH_4_][NH_2_]	265_onset_-350_peak_,297 (2:1); 400 (1:1)	8 wt.%	[311]
γ- Mg(BH_4_)_2_-0.5LiH	-	380–420	15.5 wt.%exp., 14.78 wt.% (theoretical) ^*^ ^*^ higher wt.% exp. due to usage of solvated γ- Mg(BH_4_)_2_	[315]
Ca(BH_4_)_2_–4 LiNH_2_	-	250_onset_-320_peak_ (288_mean_)	8 wt.%	[312,313]
Ca(BH_4_)_2_–4LiNH_2_-5 wt.% CoCl_2_	Co^2+^ (CoCl_2_)	150_onset_-207_peak_ (178_mean_)	>7 wt.%	[312]
Ca(BH_4_)_2_-2Mg(NH_2_)_2_	-	220–480 (270, 290 and 310–multistep)	8.3 (8.8 wt.% theoretical) not reversible (50-bar H_2_, 20–300 °C)	[309]
Ca(BH_4_)_2_-2Ca(NH_2_)_2_	-	220–480 (270, 290 and 310–multistep)	6.8 (7.5 wt.% theoretical) not reversible (50-bar H_2_, 20–300 °C)	[309]
5Ca(BH_4_)_2_-2LiBH_4_	-	83	6.7 wt.% ^*^ ^*^ theoretical	[306]
5Mg(BH_4_)_2_-2LiBH_4_	-	−29	8.4 wt.%^*^ ^*^ theoretical	[306]
2Mg(BH_4_)_2_-Ca(BH_4_)_2_	-	272, 326, 346, 398 (rehydrogenation at 288_onset_, 273_peak_)	10.5 wt.% ^*^ * theoretical	[316,317,318]
5Mg(BH_4_)_2_-Ca(BH_4_)_2_	-	−18 °C (p = 1atm) ^*^ ^*^ predicted based on DFT calculations; 150 °C	7.73 wt.%H_2_^*^ ^*^ predicted based on DFT calculations	[306]^*^ [316,317]
LiBH_4_ + 0.2 MgCl_2_ + 0.1 TiCl_3_; LiBH_4_ + 0.076 MgCl_2_ + 0.047 TiCl_3_	MgCl_2_, TiCl_3_	60_onset_-400_peak_	5 wt.%H_2_	[319,320]
LiBH_4_ + 0.09 TM oxides (TiO_2_, V_2_O_3_)	TiB_2_-possible active intermediate	200	7–9 wt.%H_2_; reversible	[319]
LiBH_4_ + 0.2 M (M = Mg, Al)	Mg/Al	60–300_fast_-600	9 wt.%H_2_; Al-based yields material probably volatile, only recharges to 3.5 wt.% capacity	[320]
LiBH_4_ + 0.0897 Al	Al	450	12.4 wt.%, partially reversible	[321]

Combined Kissinger plot and Arrhenius equation allowed establishing the first-order kinetic parameters for dehydrogenation reaction of binary borohydride–amine systems (Table 5).

The specific rate for Ca(BH_4_)_2_-2Ca(NH_2_)_2_ shows a 3.5-fold increase in reaction rate, when compared to Ca(BH_4_)_2_-2Mg(NH_2_)_2_ system. For comparison, pristine Ca(BH_4_)_2_ has slow desorption kinetics that onsets at 290° and peaks at 361 °C [309].

## 7. Model Systems for Hydrogen Storage of MBH and Other RMH (Reactive Metal Hydrides): Nanoconfinement vs. Bulk Behavior for Improved Thermodynamics and Kinetics

### 7.1. LiBH_4_

Being the lightest metal borohydride and potentially having some of the highest hydrogen storage capacity (18.4 wt.%), LiBH_4_ was first prepared in 1940 by Schlesinger and Brown by an organolithium compound and diborane B_2_H_6_ (Equation (18)) [102], and afterwards investigated in detail by many researchers [125,322,323,324,325]. Transition to a *h*-LiBH_4_ phase at 108–112°C exhibiting unexpectedly fast Li^+^ ion conductivity drove even further the pace of MBH research. Following melting (275 °C), decomposition occurs above 400 °C with an endothermic reaction enthalpy of ΔH_d_ = 74 kJ/mol [326], and leads to formation of LiH, B, and H_2_, which account for a real practical hydrogen storage of 13.8 wt.% H_2_ (Equations (79)–(81)) [327].

(79) $$\;\;\;\;o-LiBH_{4\;\left(s\right)}\overset{108-112\;{}^{\circ}C}{\Rightarrow }h-LiBH_{4\;\left(s\right)}\overset{275\;{}^{\circ}C}{\Rightarrow }LiBH_{4\;\left(l\right)}\overset{400-600\;{}^{\circ}C}{\Rightarrow }\;LiH_{\left(s\right)}+B_{\left(s\right)}+\frac{3}{2}H_{2}↑$$

(80) $$LiBH_{4\;\left(s\right)}\overset{\Delta }{\Rightarrow }\frac{5}{6}\;LiH_{\left(s\right)}+\frac{1}{12}\;Li_{2}B_{12}H_{12\;\left(s\right)}+\frac{13}{12}\;H_{2}↑\;;\;\;\;\;\;\;\;\;\;\;\Delta H=56\frac{\mathrm{kJ}}{\mathrm{mol}}$$

(81) $$LiBH_{4\;\left(s\right)}\overset{\Delta }{\Rightarrow }\;LiH_{\left(s\right)}+\frac{1}{2}\;B_{2}H_{6\;\left(g\right)}\Rightarrow \;LiH_{\left(s\right)}+B_{\left(s\right)}+\frac{3}{2}\;H_{2}↑\;\;\;\;\Delta H=125\frac{\mathrm{kJ}}{\mathrm{mol}}\;\;\;\;$$

Synchrotron radiation XRD (SR-PXD) showed the presence of many intermediate phases during decomposition of LiBH_4_ [322]. These intermediates (Equations (80) and (81)) are supported by Raman spectroscopy and ^11^B-NMR experiments [328,329]. The data provided by Zuttel et al. show that the two-step dehydrogenation of LiBH_4_ proceeds via Li_2_B_12_H_12_ formation (10 wt.% H_2_ release, ΔH = 56 kJ/mol, Equation (80)), and a highly endothermic second step leading to LiH and B, releasing 4 wt.% H_2_ (ΔH = 125 kJ/mol) [200]. A thermodynamic data scheme is pictured by Y. Yan et al., who assigned enthalpy effects to the intermediate species formation during o-LiBH_4_ decomposition [164].

The ^11^B NMR data showed B_2_H_6_ to be an impurity during dehydrogenation processes. Therefore, running the reaction of LiBH_4_ with B_2_H_6_ confirmed the formation of the hypothesized Li_2_B_12_H_12_ during the dehydrogenation pathway (Equation (82)) [330].

(82) $$2\;LiBH_{4\;}+5\;B_{2}H_{6}\overset{\Delta }{\Rightarrow }\;Li_{2}B_{12}H_{12\;}+13\;H_{2}↑$$

Attempts to lower the hydrogen desorption to temperatures below 400 °C have been successful when using SiO_2_ powder addition (LiBH_4_:SiO_2_ = 1:3) [331]. Three important peaks in TPD spectra of LiBH_4_ corroborated the NMR studies, Raman spectroscopy, and XRD data, all of which point to the formation of dodecahydro-closo-dodecaborate species [B_12_H_12_]^2−^, and possibly other boron hydrides as intermediates [328,329]. 

Rehydrogenation to reform LiBH_4_ is possible, but it requires extreme conditions (70–350-bar H_2_, t > 600 °C) and will not proceed to completion, thus leading to continual decrease in hydrogen capacity with the number of hydrogen release–uptake cycles (Equations (10) and Equation (83)) [48,326,332,333].

(83) $$LiH_{\left(s\right)}+B_{\left(s\right)}+\frac{3}{2}H_{2}\;\overset{690\;{}^{\circ}C,\;\;197.4\;atm\;H_{2}}{\Rightarrow }LiBH_{4\;\left(s\right)}$$

**Figure 15:**
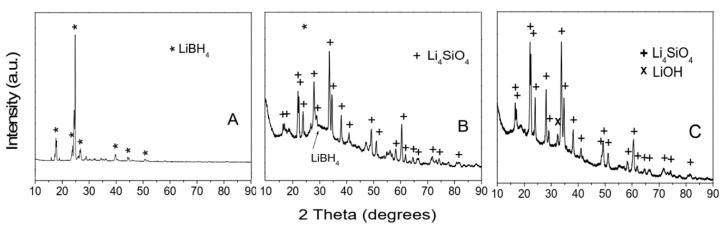
XRD data showing the evolution of phases in a Mo-catalyzed LiBH_4_-Mo:MSU-H mesoporous silica nanocomposite: commercial LiBH_4_ (**A**), rehydrogenated LiBH_4_ - Mo:MSU-H (**B**) and dehydrogenated LiBH_4_ Mo:MSU-H (**C**). Reprinted with permission from [335].

**Figure 16:**
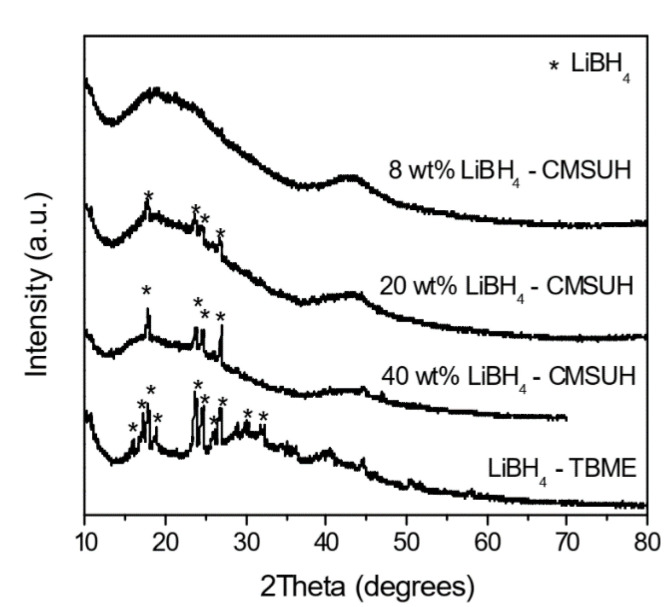
XRD diffraction spectra of MTBE-recrystallized LiBH_4_ and of three nanocomposites LiBH_4_-C-MSU- H (8 wt.%, 20 wt.%, and 40 wt.% LiBH_4_). Reprinted with permission from [334].

Nanoconfinement of LiBH_4_ was investigated in a variety of supports (2–25 nm), such as mesoporous silica (MSU-H, SBA-15 etc.) or mesoporous carbon (carbon replica of MSU-H silica: C-MSU-H, or of SBA-15 silica: C-SBA-15 i.e., CMK-3) (Figure 15) [334,335,336]. A pertinent overview of C-replica synthesis of mesoporous silicas comes from M. Kruk et al. [337] and R. Ryoo et al. [338]. Mesoporous SiO_2_, however, is to be avoided, since an irreversible reaction with LiBH_4_ during dehydrogenation could lead to lithium silicates (Figure 16) [335,339]. Attempts to improve hydrogen uptake by using Mo-decorated mesoporous silica (MSU-H type) increased the reversible hydrogen capacity from 3.2 wt.% (pristine LiBH_4_) to 5.2 wt.% H_2_ in the nanocomposite LiBH_4_-Mo:MSU-H [335].

Formation of lithium silicates (Li_2_SiO_3_, Li_4_SiO_4_) was confirmed by XRD and synchrotron powder XRD, but only after LiBH_4_ decomposed in the as-synthesized composite LiBH_4_@meso-SiO_2_; the nanocomposite seems otherwise stable at temperatures t < 275 °C (Equation (84)) [89,335,340].

(84) $$4LiBH_{4\;}+3SiO_{2}\overset{\Delta }{\Rightarrow }\;2Li_{2}SiO_{3}+Si+4B+8H_{2\;}\overset{2\;LiBH_{4}}{\Rightarrow }\frac{3}{2}Li_{4}SiO_{4}+\frac{1}{2}Si+2B+4H_{2}↑$$

Nanoconfinement can be performed by melt infiltration or solvent infiltration, both of which lead to a decrease in the crystallite size of the LiBH_4_ [341,342]. Incipient wetness technique has been used to infiltrate MTBE (methyl *tert*-butyl ether) solution of LiBH_4_ in mesoporous carbon (C-MSU-H). The maximum loading attempted was about 40 wt.% LiBH_4_, but the XRD data confirms that even for a 20 wt.% loading some diffraction peaks of LiBH_4_ appear. This is indicative of the borohydride species crystallizing outside of the mesopores [334]. 

**Figure 17:**
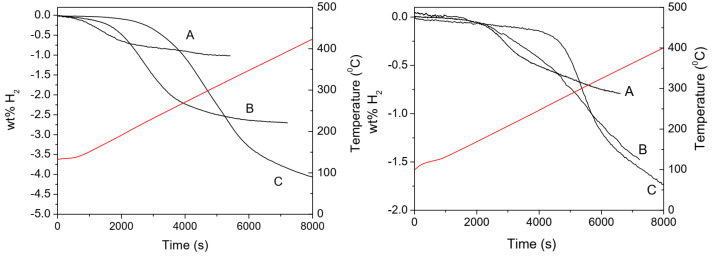
Hydrogen desorption kinetics of nanoconfined LiBH_4_@C-MSU-H with a heating ramp of 2 °C/min (initial nanocomposites, left; rehydrogenated samples, right). A: 8 wt.%, B: 20 wt.%, and C: 40 wt.% loading of LiBH_4_. Reprinted with permission from [334].

Interestingly, the lowest onset for the as-prepared nanocomposites LiBH_4_@C-MSU-H was recorded at 150 °C for the lowest investigated borohydride loading (8 wt.%), while the others showed decomposition starting at 170 °C (20 wt.% LiBH_4_) and 200 °C (40 wt.% LiBH_4_), with effect ascribed to the better dispersion of the borohydride inside the mesoporous support at lower loadings (Figure 17) [334,335].

The influence of carbon porosity was investigated showing that a proper impregnation of LiBH_4_ inside 1.8–3.2 nm activated carbon (S_BET_ = 1341 m^2^/g; V_p_ = 0.87 cm^3^/g) is accompanied by reduction of both surface area (to 245 m^2^/g) and pore volume (to 0.2 cm^3^/g) [336,343]. The size of the scaffold alters the activation energy E_a_ of dehydrogenation: non-porous graphite (146 kJ/mol LiBH_4_), 24 nm RF-CA (111 kJ/mol LiBH_4_), and 13 nm RF-CA (103 kJ/mol LiBH_4_), consistent with inverse proportionality between nanoscaffold size and activation energy E_a_ [344,345]. The Arrhenius Equation (85) shows that, taking solely into account the Ea of these three supports, one might expect the dehydrogenation rates to follow this order: k_graphite_:k_24nm_:k_13nm_ = 1:e^14.03^:e^17.20^ (at 298.15 K), and k_graphite_:k_24nm_:k_13nm_ = 1:e^11.28^:e^13.86^ (at 373.15 K).

(85) $$k=A\;e^{-\frac{E_{a}}{RT}}$$

This approximation was performed assuming the same pre-exponential Arrhenius factor A (an oversimplification), but this shows that nonporous carbon barely has any benefit from confinement, whereas the rate in nanoconfined composites is ~10^6^ times faster. Moreover, reducing the nanoscaffold from 24 nm to 13 nm affords a rate that is 23.8 times higher (at 25 °C) or 13.2 times higher (at 100 °C, a temperature considered optimal for operation of fuel cell operation). Nanoconfined LiBH_4_ shows a decrease in melting temperature of 30 °C (in 13 nm RF-CA) or 23 °C (in 24 nm RF-CA), but only when melt infiltration was employed; a modest 1 °C decrease was recorded for the solvent infiltration technique [341,346].

When a 33.67 wt.% loading of LiBH_4_ was achieved in C-SBA-15 (CMK-3 carbon, average diameter 4 nm) prepared by the concentrated solution infiltration technique, a clear reduction in temperature onset of dehydrogenation was recorded, and the composite LiBH_4_@CMK-3 exhibited complete dehydrogenation up to 400 °C [336]. The solvent infiltration approach can alter the dehydrogenation pathway, as well as the reaction kinetics; for comparison, a ball-milled sample containing LiBH_4_ affords full dehydrogenation above 450 °C [347].

While bulk dehydrogenated LiBH_4_ (LiH and B) can be rehydrogenated under harsh conditions (p, t, Equation (83)), the nanoconfined borohydride can be regenerated using 100-bar H_2_ at below 400 °C within 2 h when microporous carbon was used as scaffold. The improved recyclability of such a composite points to better reversibility when pore size decreases to a microporous range (D_pore_ < 2 nm) [341]. A comparison between nanoconfinement of LiBH_4_ in carbon and silica supports of pore radii 8–20 Å showed improvement in desorption kinetics, reversibility, and Li^+^ ionic conductivity with decreasing pore size. It was shown that similarly sized silica and carbon scaffolds produce different effects upon the melt infiltration of LiBH_4_, with silica-based support showing obvious improvements compared to carbon scaffolds, an aspect attributed to increased LiBH_4_ interfacial layer thickness “*t*” for SiO_2_ (1.94 ± 0.13 nm) compared to C-based support (1.41 ± 0.16 nm). In this regard, the Gibbs–Thomson equation correlates with the shift (depression) in melting temperature (ΔT) to interfacial effects, namely, it increases with the interface energy (Δγ: 0.053 J/m^2^ for LiBH_4_/SiO_2_ and 0.033 J/m^2^ for LiBH_4_/C system) and decreases with pore radius (r_p_) (Equation (86)) [348,349].

(86) $$\frac{\Delta T}{T_{0}}=\frac{2\Delta \gamma V_{m}}{\Delta H\left(r_{p}-1\right)}\;$$

The phase transition shift for LiBH_4_ (~114 °C for bulk) was observed at 65 °C for LiBH_4_/SiO_2_ (3.3 nm) and at 19 °C for LiBH_4_/C_m-4.9_ (of high microporosity). P. Ngene et al. assumed a core-shell model and found a direct relation for computing the enthalpy for the confined material, which could allow fine-tuning of thermodynamic parameter by adjusting pore size and interfacial thickness (Equation (87)) [350].

(87) $$\frac{\Delta H_{confined}}{\Delta H_{bulk}}={\left(1-\frac{t}{r}\right)}^{2}$$

Pore size distribution (PSD) and pore geometry should always play a decisive role in nanoconfinement results, especially for C-nanoscaffolds where microporosity percentage could be dominant [351]. The nano-synergy effect has recently made possible a reversible hydrogen capacity of 9.2 wt.% [352]. Investigation of possible side-pathways in a dehydrogenation reaction and particularly establishing the nature of intermediate species (such as Li_2_B_12_H_12_) could trigger new directions in nanoconfined borohydride materials [41,323,324,325]. 

### 7.2. LiBH_4_ + MgH_2_

Magnesium-based materials (alloys, hydrides, alanates, borohydrides, etc.) have sparked interest as promising hydrogen storage materials due to their low cost, high wt.% hydrogen storage, and interesting properties, including the much sought-after recyclability/reversibility condition for any future energy storage material [353]. Among all hydrides, MgH_2_ is very promising due to high abundance of magnesium, low cost, and reversibility behavior in hydrogenation studies, along with high hydrogen uptake capacity (7.7 wt.%) and very high energy density (9 kJ/g Mg) [354,355,356,357,358,359]. However, the desorption kinetics are slow at 300 °C and 1-bar H_2_, while the reactivity towards air (oxygen in particular) and the hydrogen desorption has a high enthalpy—thus requiring operation at high temperature [355,360]. Lowering desorption enthalpy can be achieved by introducing high-reactivity defects in the structure (high-energy ball-milling) reaction with a destabilizing element or with a catalyst [361].

Given the advantages of using the Mg-based hydrides mentioned above, inclusion of MgH_2_ in various reactive hydride composites (RHCs) has been attempted. The most studied such reactive composite is 2LiBH_4_ + MgH_2_ (11.6 wt.% hydrogen storage capacity, according to the decomposition pathway in Equation (88). While taken individually, both LiBH_4_ and MgH_2_ have unfavorably high thermal stability and slow kinetics, and their 2:1 mixture is found to produce MgB_2_ instead of the rock-stable boron B, which brought new hopes in pursuit of a viable energy-storage material (Equation (88)) [92,306,362].

(88) $$2\;LiBH_{4\left(l\right)}+MgH_{2\left(s\right)}\Leftrightarrow \;2\;LiH_{\left(s\right)}+MgB_{2\left(s\right)}+4\;H_{2}↑\;\;;\;\;\;\;\Delta H=45.7\frac{\mathrm{kJ}}{\mathrm{mol}}\;$$

This RHC has favorable thermodynamics and the formation of MgB_2_ reduces the dehydrogenation enthalpy to ΔH_(88)_ = −46 kJ/mol H_2_, which makes the reaction reversible under mild conditions (3–20-bar H_2_, 315–450 °C) [92,362,363,364,365,366,367,368,369]. Similar destabilization has been observed for the (LiBH_4_ + 2CaH_2_) system, with an endothermic enthalpy slightly less than 41.3 kJ/mol and 11.7 wt.% H_2_ storage (Equation (89)) [306].

(89) $$2\;LiBH_{4\left(l\right)}+CaH_{2\left(s\right)}\Leftrightarrow \;2\;LiH_{\left(s\right)}+CaB_{2\left(s\right)}+4\;H_{2}↑\;\;;\;\;\;\;\Delta H=41.3\frac{\mathrm{kJ}}{\mathrm{mol}}$$

Nanoconfinement of the (2LiBH_4_ + MgH_2_) system was attempted in 21 nm RF-CA porous scaffolds showing 4 wt.% reversible hydrogen storage capacity (92% of theoretical storage of the composite RHC@RF-CA, after three cycles), and it was found that decomposition in the nanoconfined state mirrors that from the bulk (Equation (88)) [346]. The hydrogen capacity is higher than that of nanoconfined MgH_2_@RF-CA_22nm_ (1.4 wt.%) [133]. 

DSC analysis of the composite 2LiBH_4_-MgH_2_ shows four endothermic peaks: 113, 267, 332, and 351 °C for the nanoconfined state in 21 nm RF-CA vs. 117, 290, 364, and 462 °C in bulk (Table 6) [346]. The hydrogen desorption kinetics are clearly improved by nanoconfinement, with MgH_2_ registering a 32 °C lowering of dehydrogenation temperature, while dehydrogenation of LiBH_4_ is lowered by a significant 111 °C.

Regardless of the kinetic improvements during recyclability studies, the high thermal stability of bulk metal borohydrides remains an obstacle in their use as hydrogen storage materials.

### 7.3. NaBH_4_

With a hydrogen storage capacity of 10.6 wt.%, NaBH_4_ is a valuable reducing reagent used in organic synthesis and decomposes at a high temperature typical of ionic compounds like metal salts (534 °C) [370,371,372,373,374]. The synthesis was reported first by Schlesinger, who reacted trimethyl borate with sodium hydride in hydrocarbon oil, at boiling point (250 °C) (Equation (90)) [375]. The sodium methoxide is hydrolyzed (NaOCH_3_ + H_2_O → NaOH + CH_3_OH) to form CH_3_OH, which allows separation of the reactive mixture, and a sodium hydroxide solution of NaBH_4_ that allows borohydride extraction using isopropylamine.

(90) $$4\;NaH+B{\left(OCH_{3}\right)}_{3}\Rightarrow \;NaBH_{4}+3\;NaOCH_{3}$$

An alternative method (Bayer process) utilizes Na_2_B_4_O_7_·7SiO_2_ with Na and 3 atm H_2_ at 400–500 °C in a one-pot synthesis [376,377]. This process allows for obtaining large quantities of NaBH_4_, but it does present the risk of explosion (the working temperature is above decomposition of NaBH_4_) and it produces large quantities of Na_2_SiO_3_ of lower market demand or value (Equation (91)).

(91) $$Na_{2}B_{4}O_{7}+16\;Na+8\;H_{2}+7\;SiO_{2}\;\overset{700\;{}^{\circ}C}{\Rightarrow }4\;NaBH_{4}+7\;Na_{2}SiO_{3}$$

Given the lower cost of Mg as compared to Na, a modified Bayer process uses MgH_2_ as reducing reagent (Equation (92)) [378,379,380].

(92) $$\frac{1}{4}Na_{2}B_{4}O_{7}+2\;MgH_{2}+\frac{1}{4}Na_{2}CO_{3}\;\overset{-2MgO,-\frac{1}{4}CO_{2}}{\Rightarrow }\;NaBH_{4}\;\overset{-2\;MgO}{⇐}\;NaBO_{2}+2MgH_{2}$$

The decomposition is different to that of LiBH_4_ analogue, due to the lower stability of NaH compared to LiH, and it leads to the formation of the parent elements (Equation (93)). The decomposition process of NaBH_4_ starts at ~240 °C and releases the greatest amount of hydrogen above 450 °C.

(93) $$NaBH_{4}\overset{534\;{}^{\circ}C}{\Rightarrow }\;Na+B+2H_{2}$$

### 7.4. Mg(BH_4_)_2_

With a high theoretical gravimetric energy storage capacity (14.8 wt.%), and reasonably low dehydrogenation enthalpy (ΔH = −39.3 kJ/mol H_2_), Mg(BH_4_)_2_ remains an attractive candidate for hydrogen storage. First-principles calculations on the ground-state crystal structure of Mg(BH_4_)_2_ confirm these energetic characteristics [381]. Decomposition shows several steps in order to finally produce MgB_2_ and H_2_ (Equation (94)). Formation of intermediate species such as MgB_12_H_12_ is also implied [382,383,384,385,386].

(94) $$\alpha -Mg{\left(BH_{4}\right)}_{2}\overset{190\;{}^{\circ}C}{\Rightarrow }\;\beta -Mg{\left(BH_{4}\right)}_{2}\underset{-3\;H_{2}\left(11.1\;wt.\%\right)}{\Rightarrow }MgH_{2}+2B\;\underset{-H_{2}\;\left(4.2\;wt.\%\right)}{\Leftrightarrow }\;MgB_{2}$$

**Figure 18:**
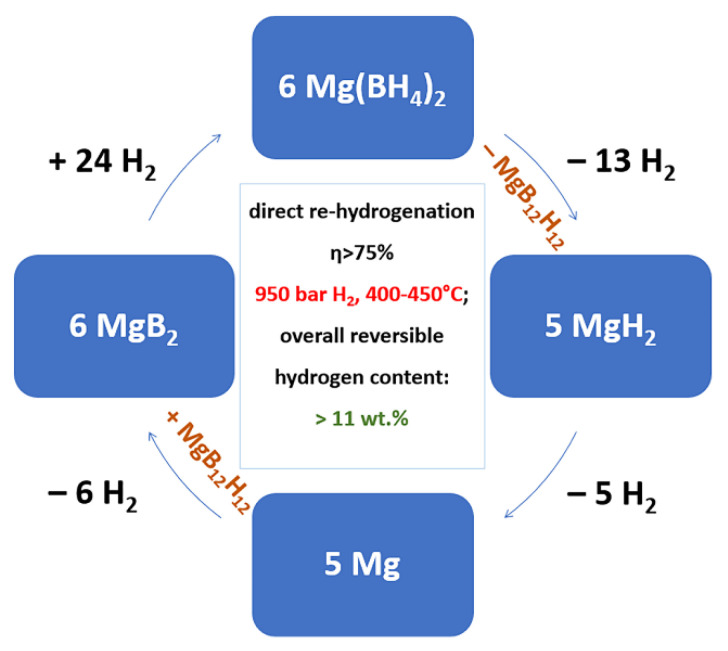
Direct re-hydrogenation of MgB_2_ under extreme conditions (950-bar H_2_, 450 °C) to regenerate Mg(BH_4_)_2_ material with ~11 wt.% reversible hydrogen capacity. Figure compiled from reaction data presented in [386].

In fact, an alternative reaction mechanism has been proposed concerning formation of the MgB_12_H_12_ species in a first step (−15 ppm, singlet, in ^11^B-MAS NMR spectrum), dehydrogenation of MgH_2_ in a second step, and a final reaction of Mg and MgB_12_H_12_ forming MgB_2_—which we know is the final dehydrogenation “resting-state” of magnesium (Figure 18).

The decomposition temperature could be lowered to 100°C by the addition of TiCl_3_ catalyst [109]. Rehydrogenation of MgB_2_ under 100-bar H_2_ at 350 °C showed ~3 wt.% hydrogen capacity, confirming the reversibility of this dehydrogenation step [156,385]. The intermediacy of MgB_12_H_12_ was investigated, as in the case of other metal borohydrides, and is considered plausible [387].

The first decomposition step (277 °C) has an enthalpy of −40 …57 kJ/mol, depending on experimental data [385,387], and a computed value of 38 kJ/mol (DFT computations) [388]. In situ synchrotron radiation SR-PXD data show as final dehydrogenation products a mixture of Mg, MgO, and MgH_2_, but these results should be considered with care given the high affinity of Mg for oxygen and proneness to subsequent oxidation [118].

Regeneration of Mg(BH_4_)_2_ by hydrogenation of MgB_2_ requires high H_2_ pressure and temperature (950-bar H_2_, 400 °C), but also prolonged time (108 h), which makes the bulk material impractical for use in a tank for mobile applications [386]. When starting from nano-sized MgB_2_ synthesized at RT, a partial rehydrogenation to Mg(BH_4_)_2_ occurred at 300°C and 30-bar H_2_, leading to a material with 2.9 wt.% hydrogen storage, but quite far off the theoretical value for magnesium borohydride (14.8 wt.%) [389,390].

Another polymorph of magnesium borohydride, γ-Mg(BH_4_)_2_, has recently shown promising results when Co-based additives were used (2 mol% CoX_2_, X = F, Cl), desorbing 4 wt.% H_2_ at 285 °C and 2.5-bar H_2_ backpressure. The reverse reaction took place at 285 °C, reforming the more stable β-Mg(BH_4_)_2_, but using 120-bar H_2_ was needed to yield a material with 2 wt.% hydrogen storage capacity. In addition, the γ → ε phase transition temperature of Mg(BH_4_)_2_ was reduced using CoX_2_ by ~50 °C [391,392]. 

Mg-based materials greatly benefit from nanoconfinement in carbon nanotubes, which reduces the enthalpy of dehydrogenation even by 50% (for MgH_2_, a reduction from 36 kJ/mol_H_ in bulk, to 17 kJ/mol_H_ in 9-unit cell thick MgH_2_ thin film slab) [393]. Nanoconfinement of Mg(BH_4_)_2_ in microporous carbon AC resulted in nanocomposites with loadings as high as 44 wt.%, albeit at the cost of partially crystallizing on the outer surface of AC (as seen by powder-XRD and EDS data analysis) [382,383]. The total weight loss of 6 wt.% corresponds to 44% borohydride loading in the nanocomposite [382]. Small angle neutron scattering (SANS) is a technique allowing for the study of particle size and distribution. Using isotopically labeled magnesium borohydride, Mg(^11^BD_4_)_2_@AC, Sartori et al. observed nanoconfined borohydride particles of less than 4 nm [383]. Improved hydrogen release is obvious from the shifting of the endothermic peak events from 269 °C to 257 °C (−12 °C decrease) and from 376 °C to 317 °C (−50 °C decrease), and it can be ascribed to the nanoconfinement effect. 

### 7.5. Ca(BH_4_)_2_

Ca(BH_4_)_2_ has a hydrogen storage capacity of 11.5 wt.% (maximum theoretical capacity), but the real capacity is only 9.6 wt.% considering the actual dehydrogenation reaction (Equations (12) and (95)). Equation (95) is essentially the same as Equation (12), but written for one mol Ca(BH_4_)_2_, as the reaction is reversible and decomposition and rehydrogenation processes follow the same reaction, having a ΔH = 32 kJ/mol [188].

(95) $$\;\alpha -Ca{\left(BH_{4}\right)}_{2}⟺\frac{2}{3}\;CaH_{2}+\frac{1}{3}CaB_{6}+\frac{10}{3}\;H_{2}\;;\;\;\;\;\;\Delta H=32\frac{\mathrm{kJ}}{\mathrm{mol}\;H_{2}}$$

The orthorhombic (*α*-phase) can be obtained free of THF solvent by evacuation under vacuum of the commercially available THF adduct. There is another low-temperature orthorhombic phase proposed for Ca(BH_4_)_2_ (γ-phase polymorph) [118]. The decomposition temperatures are higher than those anticipated based on thermodynamic data, partly due to sluggish kinetics [394]. Heating *α*-Ca(BH_4_)_2_ to 170 °C leads to a phase transition to *β*-Ca(BH_4_)_2_ and upon further heating the DSC scan data shows two endothermic peaks at 360 °C and 500 °C [395,396]. The rehydrogenation of this reversible system can proceed at 400 °C and 690 atm H_2_, but these conditions are too harsh to warrant the use of bulk Ca(BH_4_)_2_ in vehicular applications [95]. The influence of using catalysts (Nb, Ti) showed beneficial reduction in hydrogen desorption temperature, but the rehydrogenation conditions are far from ideal (350 °C, 150-bar H_2_, 12–24 h) and the re-hydrogenated material exhibits 4.5 wt.% H_2_ capacity [397]. Using additives like TiCl_3_ could lower even further re-hydrogenation kinetics, making it possible when dehydrogenated material was subjected at 350 °C for 24 h at a lower pressure of 90-bar H_2_ [394]. Ball-milling Ca(BH_4_)_2_ with 5 mol%TiCl_3_ (Ca(BH_4_)_2_:TiCl_3_ = 1:0.05) leads to hydrogen evolution and formation of CaCl_2_, according to Equation (96) [394].

(96) $$\;7Ca{\left(BH_{4}\right)}_{2}+4TiCl_{3}\Rightarrow \;6CaCl_{2}+CaB_{6}+4TiB_{2}+28\;H_{2}$$

This is also an example where the additive (TiCl_3_) alters the dehydrogenation pathway (commonly accepted as described by Equation (95)), as the proposed weight-loss based on DSC and TGA curves conform to about 7.1 to 8.7% reduction. This is consistent with the following decomposition pathway, producing CaH_2_, B, and H_2_ (7.2 wt.% hydrogen storage capacity based on Equation (97)) [394]. The hydrogen storage capacity after rehydrogenation is only 3.8 wt.%; complete rehydrogenation was thus not achieved. It is, however, apparent that TiCl_3_ additive plays an important role in lowering activation energy for the hydrogenation reaction.

(97) $$\;Ca{\left(BH_{4}\right)}_{2}\Rightarrow \;CaH_{2}+2\;B+3\;H_{2}$$

A proof-of-concept process for complete hydrogen release/uptake was shown for a ball-milled mixture CaB_6_ + 2 CaH_2_ + 8 wt.% TiCl_3_/Pd, but the reaction conditions were harsh (700-bar H_2_, 400–440 °C); however, almost complete recyclability with 9.6 wt.% hydrogen storage has been demonstrated [95]. A key role in this achievement is thought to be played by the deposition of TiCl_3_ on porous Pd, which is itself known for absorbing record amounts of hydrogen and giving interstitial hydrides, while other TM halides (RuCl_3_) led to polymorphs of Ca(BH_4_)_2_ and other unknown phases, confirming the alteration in the rehydrogenation pathway [95,398].

**Figure 19:**
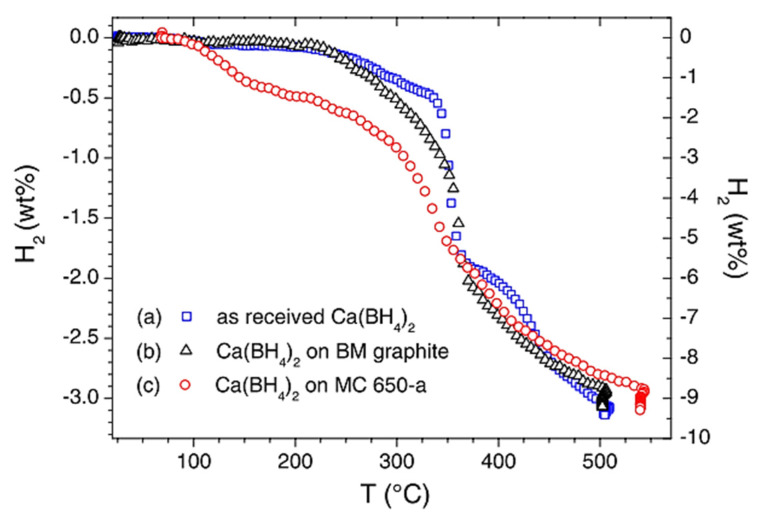
TPD curves of Ca(BH_4_)_2_@MC-a nanocomposites with a heating rate of 2.5 °C/min. Reprinted with permission from [143].

Nanoconfined Ca(BH_4_)_2_ in microporous carbon showed tangible improvements in the recyclability behavior of as-prepared nanocomposites [143]. Using carefully engineered chemically activated micro-mesoporous carbon as scaffolds (specific surface area SSA = 1780 m^2^/g, V_pore_~1 cm^3^/g and 60% microporosity), Comanescu et al. obtained by the incipient wetness method Ca(BH_4_)_2_@MC-a nanocomposites with 36 wt.% borohydride loading (Figure 19). 

**Figure 20:**
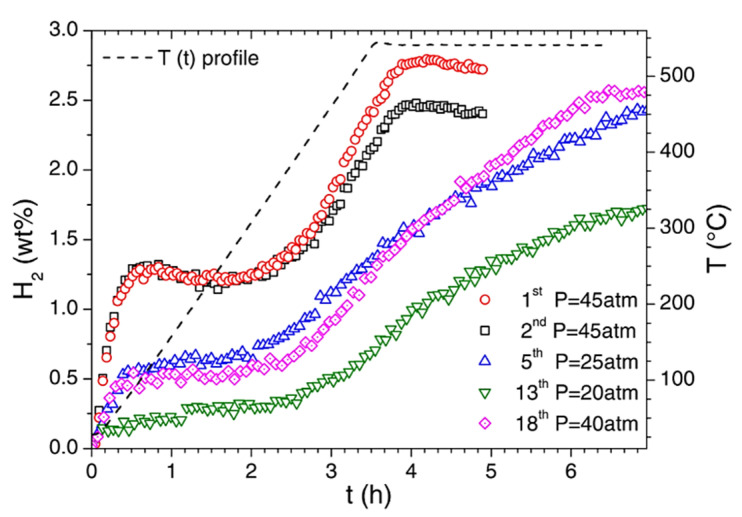
TPA curves of nanocomposite Ca*.(*BH_4_)*/*_2_ *=* MC 650-a for successive hydrogenation cycles at pressures in the range 20–45 atm. Reprinted with permission from [143].

These Ca(BH_4_)_2_@MC-a composites started the hydrogen release step at about 100 °C and managed to retain 2.4 wt.% hydrogen capacity even after 18 cycles of hydrogen release/uptake. The rehydrogenation of the desorbed composites needed rather mild conditions (25–40 atm H_2_, 6.5 h), confirming the important role of the nanoporous scaffold of choice (Figure 20) [143]. 

The result obtained for the composite Ca*.(*BH_4_)*/*_2_ *=* MC 650-a (containing 36 wt.% Ca(BH_4_*)*_2_*)* is an improvement over previously reported data on reversibility studies on Ca(BH_4_)_2_, confirming that about 68% of Ca(BH_4_)_2_ behaves reversibly [143].

### 7.6. Ammonia Borane NH_3_BH_3_

A special place is reserved to ammonia–borane, an interesting adduct with 19.6 wt.% hydrogen. NH_3_BH_3_ (ammonia–borane, AB) and ammonium borohydride (NH_4_BH_4_) have been reported from 1958, but NH_4_BH_4_ is far too unstable releasing H_2_ at even −40 °C to form AB [399]. NH_3_BH_3_ is a stable solid, water-sensitive, and susceptible to oxidation or attack by acidic reagents, having a high solubility in water (33.6 g/100 g H_2_O). The AB molecules present dipole–dipole interactions due to the dihydrogen bonding, i.e., interaction between a hydridic hydrogen (-NH_3_) and an acidic one (BH_3_) [400], and organizes in an orthorhombic phase at low temperature (<225 K) due to these interactions. While it is not a proper metal borohydride, NH_3_BH_3_ has the advantageous property of being RT-stable, exhibiting a two-step dehydrogenation at very reasonable temperatures: 114 °C (melting point, 6.5 wt.% H_2_ release, forming polyaminoboranes (NH_2_BH_2_)_n_), and 150 °C (remaining H_2_ release, formation of polyiminoborane (NHBH)_n_), with the downsides of irreversibility, slow kinetics, and formation of “inorganic benzene” as by-product during dehydrogenation (borazine, *c-*(NHBH)_3_) (Equation (98)). The N-B (nitrogen–boron) units are isoelectronic to C-C (carbon–carbon) moieties, and they can therefore be regarded as hydrocarbon analogues [145,401,402].

(98) $$n\;NH_{3}BH_{3}\overset{70-120\;{}^{\circ}C,-nH_{2}}{\Rightarrow }{\left(NH_{2}BH_{2}\right)}_{n}\;\overset{120-200\;{}^{\circ}C,-nH_{2}}{\Rightarrow }{\left(NHBH\right)}_{n}\overset{>500\;{}^{\circ}C,-nH_{2}}{\Rightarrow }\;{\left(BN\right)}_{n}$$

Mesoporous silica or carbon have served as scaffolds for NH_3_BH_3_ impregnation, and this nano-reduction strategy bypassed borazine formation [145,402,403]. Even NH_3_ release is suppressed when Li^+^-based dopants are used [402]. Some key thermodynamic aspects connected to NH_3_BH_3_ confinement in nanoporous substrates are summarized in Table 7. A common denominator is that using lower-size pores, the decomposition temperature is decreased due to the lowered activation energy *Ea* caused by nanoconfinement [404,405].

The porosity of the MOF scaffolds (IRMOF-1, IRMOF-10, UiO-66, UiO-67, MIL-52(Al)) for AB nanoconfinement (8.38 wt.%, 3.51 wt.%, 12.95 wt.%, 14.70 wt.%, and 11.26 wt.%, respectively, computed from SI data) has been investigated, showing that AB impregnation does not change MOF crystallinity. A low desorption temperature of 64°C was recorded for AB@UiO-66 (12.95 wt.% loading), which also featured the smallest pore size among investigated MOFs, of only 1.17 nm [411]. 

The reduction in decomposition temperature of AB@MOF was found to be linearly proportional to the reciprocal of AB size; in other words, the expectations are that a lower pore size nano-restriction would yield the best destabilization in ammonia borane nanocomposites [411].

Another advantage of utilizing SiO_2_-based scaffolds in AB nanoconfinement is that this does not lead to a reaction with the framework, as was the case for typical M(BH_4_)_x_. It is also apparent from Table 7 that 1D-structured mesosilica (MCM-41) are less attractive as scaffolds than 2D-mesostructured silica (SBA-15) of hexagonal pore arrangement, although the pore size might suggest otherwise (4 nm for MCM-41 vs. 7.5 nm for SBA-15). These conclusions could be extended to other borohydride@silica nanosystems as well, and could provide a starting point for further nanoscaffold engineering research. Nanoconfinement alters the dehydrogenation pathway in AB, as utilizing mesoporous carbons like CMK-3 manages to avoid toxic gas evolution; borazine or ammonia are not detected in that case. It can be hypothesized that nanoconfinement might help rehydrogenation by ammonia borane restructuring inside scaffold nanopores, and by lowering activation energies to improve the thermodynamics of hydrogen release/uptake. Some advances have been made by suggesting a methanolysis cycle, where [NH_4_][B(OCH_3_)_4_] and B(OCH_3_)_3_ are key intermediates [412]. Complete elucidation of a yet-unclear dehydrogenation mechanism, combined with collaborative theoretical/experimental research efforts, could pave the way to the ultimate hydrogen-storage material. 

## 8. Conclusions and Outlook

It has been almost half a century since hydrogen was first considered a renewable energy carrier, aiming at replacing fossil fuel and building up towards a “hydrogen economy” goal. While not without its pitfalls, solid-state hydrogen storage exhibits obvious advantages compared to the physical storage of H_2_ gas: it is safer, requires less extreme conditions for storage and regeneration, and occupies less space. 

Among various metal hydrides, metal borohydrides have inherited structural flexibility, accommodating both mixed cations and anion substitutions, and can be tailored towards multi-functional materials. Borohydrides can be engineered to form novel composites that reversibly store hydrogen chemically, reaching very good hydrogen storage capacities, in line with the DOE’s expectations for the near future. Recent studies conducted under high temperature and pressure (hydrogen, diborane) have shed new light on mechanistic aspects regarding formation and decomposition pathways of complex metal borohydrides. 

Very recent progress on alkali metal borohydrides is directed towards controlling the kinetics of hydrogen production by utilizing nanoporous silica and carbon, Co-based catalysts, bimetallic catalysts (NiPt, NiCo_9_, RuPd, Co-Ru) and nano-catalyst composites, or harnessing the latent potential in alcoholysis (methanolysis, in particular) of said metal borohydrides. Kinetic studies on metal borohydrides currently target PEM fuel cell applications, and have shown that the hydrolysis mechanism has as a rate-limiting step the splitting of the H-OH bond. Transition metal nanoparticles (Ni, Pt, Zr, Co, Mn, Cu), mixed-valence oxides (Co_3_O_4_), transition metal complexes (of Ru^3+^, for instance), and MOFs have shown controlled hydrolysis of alkali metal borohydrides. Engineered catalysts (Mg_95_Ni_5_) have managed to suppress ammonia release from borohydride ammoniates by improving hydrogen release at very reasonable temperatures (70–80°C). Exploring new decomposition pathways of such ligand-stabilized borohydrides has also gained new momentum. Divalent metal borohydrides stabilized by neutral molecules show high ionic conductivity and a real promise for use in solid-state batteries. The rich boron–hydrogen chemistry, together with the open channels of closo-structured borohydrides (B_10_H_10_^2−^, B_12_H_12_^2−^), currently make research on closo-borate salts of alkali and alkali-earth metals a hot topic in ionic conductivity for solid-state electrolytes. Direct borohydride fuel cell (DBFC) and direct borohydride–hydrogen peroxide fuel cell (DBHPFC) systems research has expanded recently towards high-performance applications aimed at space exploration, by harnessing the power of nanoparticle catalysis (Ni, Cu, or bimetallic PdFe, PdAg, PdAu) to control the resultant power density. Various destabilization strategies of borohydride compounds have now included novel catalyst systems and complementary in-depth mechanistic insights. Nanostructuring of employed catalysts for hydrolysis reactions or de-/redrydrogenation studies has confirmed a positive effect by lowering activation energies associated with individual mechanistic steps. The porous polymorph of Mg(BH_4_)_2_ has gained attention recently by involvement in a core-shell, oxidation-resistant nanostructure (γ-Mg(BH_4_)_2_@MgCl_2_) to afford dehydrogenation onset as low as 100 °C. Theoretical computations on ternary borohydride materials (K_2_B_2_H_8_) predict superconducting properties at high pressures and around 140 K. Fundamental research is constantly expanding the class of structurally characterized metal borohydrides with new members (Th(BH_4_)_4_ is a new addition from 2021), while more cost-effective and high-yielding synthesis routes emerge—the synthesis of NaBH_4_ was reported by milling sodium tetraborate with Al and NaH as additive.

Meeting targets related to performance range, refueling time, car performance, and passenger space and safety are at the forefront of development of novel hydrogen storage materials and material-based storage system technologies. Mobile applications and vehicular applications in particular could receive a new breath of life when a proper hydride-based material is engineered by scientists, in line with safety, performance, and cost estimation goals.

## Figures and Tables

**Table 1:** Selected metal borohydrides with corresponding space group, decomposition temperature (and rehydrogenation temperature, where available) (°C), and crystal system.

Metal Borohydride	Decomposition Temperature (°C)	Rehydrogenation Temperature (°C)	Space Group	Crystal System	Reference
o-LiBH_4_	(mp = 277);	600	*Pnma*	Orthorhombic	[30,31,32,33,34,35,36,37,38,164]
h-LiBH_4_	(107)	600	*P6_3_mc*	Hexagonal	[30,31,32,33,34,35,36,37,38]
α-NaBH_4_	535	270–400 (2NaH/ MgH_2_)	$Fm\bar{3}m$	Cubic	[165,166,167,168,169]
Be(BH_4_)_2_	(mp = 91.3) t_d_ = 123	Explosive decomp. in air/moisture	*I4_1_/cd*	Tetragonal; helical polymeric chains BeH_2_BH_2_BeH_2_BH_2_ and terminal bidentate BH_4_ groups	[170]
α-Mg(BH_4_)_2_ (6 known polymorphs)	~300	(390 °C, 90-bar H_2_, 72 h) (400 °C, 80-bar D_2_) RT stable	*P6_1_22*	Hexagonal	[106,171]
β-Mg(BH_4_)_2_	~300	HT polymorph; RT metastable	*Fddd*	Orthorhombic	[172,173]
γ-Mg(BH_4_)_2_	~300	RT metastable	*Ia* 3 *d*	Cubic; 3D network of interpenetrated channels (1st borohydride with permanent porosity, S = 1505 m^2^/g)	[158]
α-Ca(BH_4_)_2_ (4 known polymorphs)	347–387 °C, 397–497 °C (2-step process)	RT stable	*F2dd*	Orthorhombic	[146,147,148,149,150,151]
β-Ca(BH_4_)_2_	$\alpha -\mathrm{Ca}({\mathrm{BH}}_{4}{)}_{2}\;\overset{167\;{}^{\circ}C}{\Rightarrow }$ β-Ca(BH_4_)_2_	RT, metastable, HT polymorph	$P\bar{4}$	Tetragonal	[146,147,148,149,150,151,157]
α-Al(BH_4_)_3_	<100	−123	*C2/c*	Monoclinic	[83,84,85,86,87,88]
β-Al(BH_4_)_3_	<100	−78	*Pna2_1_*	Orthorhombic	[83,84,85,86,87,88]
α-Y(BH_4_)_3_ (β-Y(BH_4_)_3_ is the HT polymorph)	190, 270	180 °C (α-to-β phase transition)	$Pa\bar{3}$	Cubic	[67,68,69,70,71,72,73]
α-Mn(BH_4_)_2_ (4 known polymorphs)	130	RT stable	*P3_1_12*	Hexagonal	[174,175,176,177,178,179,180]
LiSc(BH_4_)_4_	400	(400), irreversible hydrogen storage in Li-Sc-B-H system	$P\bar{4}$ *2c*	Tetragonal	[181,182,183]
NaZn(BH_4_)_3_	>85 °C (at 110 °C NaBH_4_ reacts with Na_2_ZnCl_4_ to form Zn and NaCl)	stable RT	*P2_1_c*	Monoclinic	[41,184]
NaZn_2_(BH_4_)_5_	unstable, converts at low temp. to NaZn(BH_4_)_3_	unstable at RT or −32 °C	*P2_1_c*	Monoclinic	[41,184]

**Table 2:** Ion conductivity $\sigma_{M^{n+}}\;\left(\frac{S}{cm}\right)$ of representative alkali and alkali-earth metal borohydrides.

Compound	$\mathrm{Ion}\;\mathrm{Conductivity}\;\sigma_{M^{n+}}\;\left(\frac{S}{cm}\right)$	Experimental Conditions/Observations	Reference
LiBH_4_	2 ∙ 10^−3^	107 °C	[261]
LiBH_4_·H_2_O	4.89 ∙ 10^−4^	t = 45 °C	[264]
LiBH_4_·x NH_3_	2.21 ∙ 10^−3^	0 < x < 2; σ=σ(x); $\sigma_{max}@40\;{}^{\circ}C$	[265,266]
LiBH_4_·x NH_3_@Li_2_O	5.4 ∙ 10^−4^	0.67 < x < 0.8; 20 °C; max for 78 wt.% Li_2_O	[267]
LiBH_4_·x NH_3_BH_3_	1.47 ∙ 10^−5^–4.04 ∙ 10^−4^	½ < x < 1; highest for x = 1	[268]
LiBH_4_·NH_3_BH_3_	10^−1^	t = 55 °C; *record value* for Li^+^	[263]
Li_2_B_12_H_12_	10^−1^	110 °C	[257]
Li_2_B_10_H_10_	3 ∙ 10^−2^	81 °C	[258]
Na_2_B_10_H_10_	3 ∙ 10^−2^	RT	[258]
Na_2_B_12_H_12_	10^−1^	256 °C	[259]
LiBH_4_-nano	10^−3^	RT; nanoconfined electrolyte	[260]
Mg(BH_4_)_2_	<10^−12^	t = 30 °C	[269]
Mg(BH_4_)(NH_2_)	10^−6^	t = 150 °C	[270]
Mg_3_(BH_4_)_4_(NH_2_)_2_	4.1 ∙ 10^−5^	100 °C	[271]
Mg(BH_4_)_2_·*en* *en* = NH_2_CH_2_CH_2_NH_2_	6 ∙ 10^−5^	70 °C, crucial role of [BH_4_^−^], Mg(en)_2_X_2_ showed σ very low	[268,272]
Mg(BH_4_)_2_·1/2*dy* *dy* = diglyme	2 ∙ 10^−5^	80 °C; chelating of flexible *dy* ligand	[273]
Mg(BH_4_)_2_·x NH_3_	3.3 ∙ 10^−4^	x = 1,2,3 6; t = 80 °C; *pas-de-deux* mechanism proposed	[274]
Mg(BH_4_)_2_·1.6NH_3_	2.2 ∙ 10^−3^	t = 55 °C	[275]

**Table 3:** Thermodynamic parameters characteristic to metal borohydrides: ΔH*_boro_*, T*_d_*, and t_d_. In brown are shown elements with $\chi_{P}\geq 1.57$ (ΔH > 0, unstable); decomposition temperature is given both as T_d_(K) and t_d_(°C), for easier evaluation.

Metal (M)	M(BH_4_)_x_	$\chi_{P}$	$T_{d}=175.914{\left(\chi_{P}-3.11\right)}^{2}\;[K]$	*t_d_* [°C]	$\Delta H_{boro}=248.7\;\chi_{P}-390.8$ $\left[\frac{\mathrm{kJ}}{\mathrm{mol}\;{\mathrm{BH}}_{4}}\right]$	Reference
Li	LiBH_4_	0.98	798.1	524.95	−147.074	[30,31,32,33,34,35,36,37,38,196,199]
Na	NaBH_4_	0.93	836.01	562.86	−159.509	[165,168,169]
K	KBH_4_	0.82	922.51	649.36	−186.866	[165,166,168]
Cu	CuBH_4_	1.9	257.56	−15.59	81.73	[113] forms at 253 K, dec.at 261–273 K
Rb	RbBH_4_	0.82	922.51	649.36	−186.866	[167]
Ag	AgBH_4_	1.93	244.94	−28.21	89.191	[114]; forms at 193 K, dec. at 243 K
Cs	CsBH_4_	0.79	946.84	673.69	−194.327	[165]
Au^I^	AuBH_4_	2.54	57.15	−216	240.898	[115] * unstable
Au^III^	Au(BH_4_)_3_	2.54	57.15	−216	240.898	[116]
Be	Be(BH_4_)_2_	1.57	417.2	144.05	−0.341	[170]
Mg	Mg(BH_4_)_2_	1.31	569.96	296.81	−65.003	[106,158,171,172,173,189,190,191,192,193,194,195]
Ca	Ca(BH_4_)_2_	1	783.19	510.04	−142.1	[146,147,148,149,150,151,157]
Cr	Cr(BH_4_)_2_	1.66	369.86	96.71	22.042	[28,29]
Mn	Mn(BH_4_)_2_	1.55	428.1	154.95	−5.315	[175,189,190,191,224]
Fe	Fe(BH_4_)_2_	1.83	288.22	15.07	64.321	[192,193]
Ni	Ni(BH_4_)_2_	1.91	253.32	−19.83	84.217	[195] * as heteroleptic complex
Co	Co(BH_4_)_2_	1.88	266.14	−7.01	76.756	[194] * presumed, but unstable
Zn	Zn(BH_4_)_2_	1.65	374.98	101.83	19.555	[119,120,121,122]
Sr	Sr(BH_4_)_2_	0.95	820.74	547.59	−154.535	[147,148]
Ba	Ba(BH_4_)_2_	0.89	866.97	593.82	−169.457	[148]
Cd	Cd(BH_4_)_2_	1.69	354.71	81.56	29.503	[149]
Hg	Hg(BH_4_)_2_	2	216.74	−56.41	106.6	[151] * failed attempts
Al	Al(BH_4_)_3_	1.61	395.81	122.66	9.607	[83,84,85,86,87,88]
Sc	Sc(BH_4_)_3_	1.36	538.74	265.59	−52.568	[182,183,295,296,297,298,299]
Ti^III^	Ti(BH_4_)_3_	1.54	433.61	160.46	−7.802	[176,177,178,179,206] * Ti(IV) is reduced in situ to Ti(III)
V	V(BH_4_)_3_	1.63	385.32	112.17	14.581	[66]
Ga	Ga(BH_4_)_3_	1.81	297.29	24.14	59.347	[84,85] * as GdH(BH_4_)_2_ (volatile, 203K) and GdH_2_(BH_4_) (unstable)
Y	Y(BH_4_)_3_	1.22	628.38	355.23	−87.386	[65,66,68,69,70,71,72,73]
Nb	Nb(BH_4_)_3_	1.6	401.1	127.95	7.12	[180] * no homoleptic form; as complex
In	In(BH_4_)_3_	1.78	311.17	38.02	51.886	[86]
La	La(BH_4_)_3_	1.1	710.71	437.56	−117.23	[212]
Ce	Ce(BH_4_)_3_	1.12	696.64	423.49	−112.256	[212]
Nd	Nd(BH_4_)_3_	1.14	682.7	409.55	−107.282	[69]
Sm^II^	Sm(BH_4_)_2_	1.13	689.65	416.5	−109.769	[213,214]
Sm^III^	Sm(BH_4_)_3_	1.13	689.65	416.5	−109.769	[214]
Pr	Pr(BH_4_)_3_	1.13	689.65	416.5	−109.769	[64]
Eu^II^	Eu(BH_4_)_2_	1.2	641.75	368.6	−92.36	[213]
Eu^III^	Eu(BH_4_)_3_	1.2	641.75	368.6	−92.36	[70,148]
Gd	Gd(BH_4_)_3_	1.2	641.75	368.6	−92.36	[68,71,72,214]
Tb	Tb(BH_4_)_3_	1.22	628.38	355.23	−87.386	[73,214]
Dy	Dy(BH_4_)_3_	1.23	621.75	348.6	−84.899	[68]
Ho	Ho(BH_4_)_4_	1.24	615.15	342	−82.412	[69]
Er	Er(BH_4_)_3_	1.24	615.15	342	−82.412	[214]
Tm	Tm(BH_4_)_3_	1.25	608.59	335.44	−79.925	[73]
Yb^II^	Yb(BH_4_)_2_	1.1	710.71	437.56	−117.23	[215]
Yb^III^	Yb(BH_4_)_3_	1.1	710.71	437.56	−117.23	[215]
Lu	Lu(BH_4_)_3_	1.27	595.57	322.42	−74.951	[73]
Th	Th(BH_4_)_4_	1.3	576.31	303.16	−67.49	[78,206,207]
Pa	Pa(BH_4_)_3_	1.5	455.99	182.84	−17.75	[78]
U	U(BH_4_)_4_	1.38	526.49	253.34	−47.594	[78,80,208,209]
Np	Np(BH_4_)_4_	1.36	538.74	265.59	−52.568	[79,80,210]
Pu^III^	Pu(BH_4_)_3_	1.28	589.12	315.97	−72.464	[78,80]
Pu^IV^	Pu(BH_4_)_4_					[79]
Tl^I^	TlBH_4_	1.62	390.55	117.4	12.094	[87]
Tl^III^	Tl(BH_4_)_3_	1.62	390.55	117.4	12.094	[88] * failed attempts; as TlCl(BH_4_)_2_ (dec. at 178 K)
Ge	Ge(BH_4_)_4_	2.01	212.86	−60.29	109.087	no conclusive reports
Zr	Zr(BH_4_)_4_	1.33	557.37	284.22	−60.029	[203,204]
Sn	Sn(BH_4_)_4_	1.96	232.65	−40.5	96.652	no conclusive reports
Hf	Hf(BH_4_)_4_	1.3	576.31	303.16	−67.49	[205]

**Table 5:** Kinetic parameters for dehydrogenation of binary destabilized systems Ca(BH_4_)_2_-M^II^(NH_2_)_2_ (M = Mg, Ca).

Binary System	Ea (kJ/mol)	A × 10^−9^ (min^−1^)	k × 10^2^ (min^−1^)	Reference
Ca(BH_4_)_2_-2Mg(NH_2_)_2_	132.7	15	1.2	[309]
Ca(BH_4_)_2_-2Ca(NH_2_)_2_	119.3	3.2	4.3	[309]

**Table 6:** DSC analysis data for RF-CA-nanoconfined 2LiBH_4_-MgH_2_ vs. 2LiBH_4_-MgH_2_-bulk [346].

Compound	DSC Peak 1; Polymorphic Transformation *o*-LiBH_4_→*h*-LiBH_4_	DSC Peak 2; Melting Point	DSC Peak 3; DehyDrogenation (MgH_2_)	DSC Peak 4; DehyDrogenation (LiBH_4_)
2LiBH_4_-MgH_2_@RF-CA_21nm_	113 °C	267 °C	332 °C	351 °C
2LiBH_4_-MgH_2_-bulk	117 °C	290 °C	364 °C	462 °C

**Table 7:** Nanoconfinement of ammonia borane in nanoporous silica (MCM-41, SBA-15) or carbon (CMK-3 OMC, RF-CC).

Substance	Scaffold	Scaffold Pore	Loading (wt.%)	t_d_ (°C)	Ea (kJ/mol)	ΔH (kJ/mol)	Ref.
NH_3_BH_3_	-	-	100	114 (110), 155	184	−19.6 to −21	[145,406]
NH_3_BH_3_	MCM-41	4 nm	33, 50, 75 (outside pores)	100, 182	N/A	34.09 (2 steps: 30.33 and 3.76)	[404,407]
NH_3_BH_3_	SBA-15	7.5 nm	50	50–100, 130	67	−1	[145]
NH_3_BH_3_	OMC	4.5 nm		(50-)95		−2.1	[402]
NH_3_BH_3_	RF-CC (carbon cryogel)	2–20 (mean 5) nm	24	85–150; 80–90 for CC(exo- peak)	N/A	−120 (CC)	[403,408]
NH_3_BH_3_	AC (activated carbon)	3 nm	17.6 wt.% (computed from pore volume decrease from 0.36 cm^3^/g to 0.14 cm^3^/g for AC@AB_THF_)	3–4; RT(25)	N/A	−1.6 (AC@AB_THF_), −3.0 (AC@AB_MET_) and −5.9 (AC@AB_milled_)	[409]
NH_3_BH_3_	MOF (Fe–MIL-53)	11–13 nm	AB:Fe = 6.5% (0.5:1 molar); 13% (1:1 molar)	80–110	130 +/− 7; 135 +/−3	N/A	[410]

## Data Availability

Not applicable.
